# *Peri*‐Alkylated Terrylenes and Ternaphthalenes Building‐Blocks Towards Multi‐Edge Nanographenes**

**DOI:** 10.1002/chem.202401462

**Published:** 2024-06-03

**Authors:** Vikas Sharma, Hassan Khan, Michael Walker, Hamid Ahmad, Anmol Thanai, Tomasz Marszalek, Dieter Schollmeyer, Martin Baumgarten, Emrys W. Evans, Ashok Keerthi

**Affiliations:** ^1^ Department of Chemistry School of Natural Sciences The University of Manchester Oxford Road Manchester M13 9PL UK; ^2^ Department of Chemistry Swansea University Singleton Park Swansea SA2 8PP UK; ^3^ Centre for Integrative Semiconductor Materials Swansea University Fabian Way Swansea SA1 8EN UK; ^4^ Max Planck Institute for Polymer Research Ackermannweg 10 Mainz D-55128 Germany; ^5^ Department of Chemistry Johannes Gutenberg-University Mainz Duesbergweg 10–14 55128 Mainz Germany; ^6^ National Graphene Institute The University of Manchester Manchester M13 9PL UK

**Keywords:** *Bay*-extension of Terrylene, Multi-edge Nanographenes, *Peri*-alkylation of Rylene, Terrylene, Transient Absorption Spectroscopy

## Abstract

Since its first synthesis by Clar in 1948, terrylene – a fully connected ternaphthalene oligomer *via* naphthalene's *peri*‐positions – has gained special focus within the rylene family, drawing interest for its unique chemical, structural, optoelectronic and single photon emission properties. In this study, we introduce a novel synthetic pathway that enhances the solubility of terrylene derivatives through complete *peri*‐alkylation, while also facilitating extensions at the *bay*‐positions. This approach not only broadens the scope of terrylene's chemical versatility but also opens new avenues for developing solution processable novel multi‐edge nanographenes and tailoring electronic energy levels through topological edge structures. Our findings include a comprehensive structural and spectroscopic characterization along with transient absorption spectroscopy and photophysics of both the synthesized *peri*‐alkylated terrylene and its phenylene‐fused derivative.

## Graphical abstract

A facile synthetic route has been developed to produce highly soluble *peri*‐alkylated terrylene and its derivatives with enhanced π‐conjugation at bay‐regions. The synthesized terrylene derivatives exhibit remarkably high fluorescence quantum yields and molar extinction coefficients, making them ideal candidates for applications in single‐molecule spectroscopy, photonics, and quantum emission.

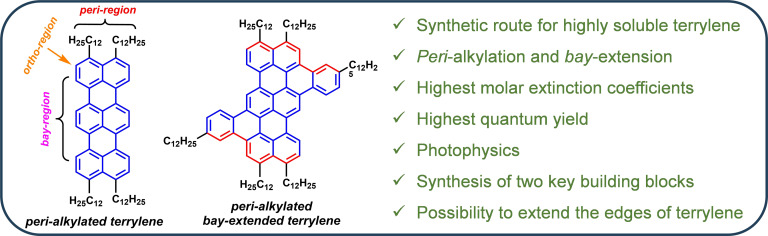

## Introduction

Rylenes, as defined by Clar, refers to poly(*peri*‐naphthalene), a specific class of polycyclic aromatic hydrocarbons (PAHs).^[1]^ They are derived by connecting all the *peri*‐positions of naphthalene units in the oligo(*peri*‐naphthalene) and result in perylene, terrylene, quaterrylene, pentarylene, hexarylene, and so on, based on the number of naphthalene units present in the oligomer starting from two.^[2]^ The higher order rylenes are considered as ultra‐narrow armchair‐edge graphene nanoribbons (AGNRs) and predicted to be either metallic or semiconducting with a very small band gap.[^3^, ^4^] The *bay*‐extended rylenes can lead to multi‐edge nanographenes (NGs) and graphene quantum dots.[^5^, ^6^, ^7^, ^8^] Perylene, first in the series of rylene family, has been extensively investigated in terms of chemical, photophysical and optoelectronic properties resulting in various applications from biology to materials science and engineering[^9^, ^10^, ^11^, ^12^] since its first synthesis by Scholl in 1910.^[13]^ However, terrylene and other higher order rylenes are comparatively less explored due to the challenges in chemical synthetic routes, solubility, and purification methods.^[14]^

**Figure 1:**
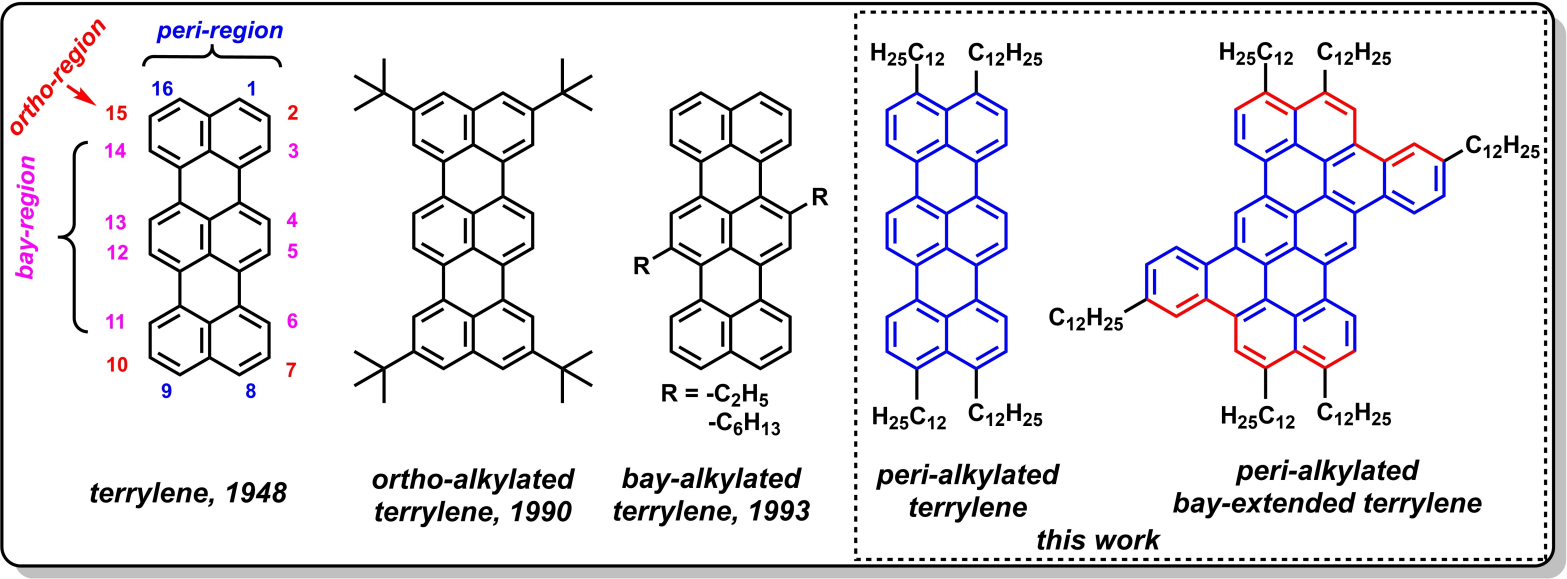
Chemical structures of terrylene and alkylated at *ortho*‐, *bay*‐ and *peri*‐regions along with *bay*‐extended phenylene‐fused terrylene.

First synthesis of terrylene was reported by Clar in 1948^[1]^ and then there were only few reports on synthesis and characterization of terrylene.[^2^, ^15^] Until late 1989, there were almost no efforts devoted to synthesis due to the poor solubility and challenges with purification of terrylenes and higher order rylenes. In 1990, Müllen and colleagues had first synthesized solution processable terrylene and other oligo‐rylene derivatives.^[16]^ Various synthetic routes were developed by Müllen and colleagues to solve the solubility and characterization issues of terrylene derivatives through introducing *tertiary*‐butyl groups at the *ortho*‐positions,[^16^, ^17^, ^18^, ^19^] alkyl chains (*n*‐ethyl, *n*‐hexyl) at *bay*‐positions[^17^, ^20^] and oxidative cyclodehydrogenation of perylene and naphthalene to yield relatively pure unsubstituted terrylene^[21]^ (Figure 1). There were other reports on partial *peri*‐methyl substituted terrylene,^[22]^ fluoro‐terrylene,^[23]^ benzo‐fused terrylenes,^[24]^ *ortho*‐borylation of terrylene,^[25]^ *bay*‐aryloxy substituted terrylene and azaterrylenes.^[26]^ Comparatively, chemistry of terrylene imides was well explored due to their applications in photovoltaics, dyes, n‐type materials and organic electronics.[^27^, ^28^, ^29^, ^30^, ^31^, ^32^, ^33^, ^34^] In recent decades, there has been enormous growth in development of higher order rylenes and interest in exploration of exciting photo physical properties.[^14^, ^35^, ^36^, ^37^] For example, terrylene and dibenzoterrylene have become interesting target molecules for applications in single‐molecule spectroscopy, photonics, quantum emission, and photophysics.[^37^, ^38^, ^39^, ^40^, ^41^, ^42^] However, there appeared no reports of fully *peri*‐alkylated terrylenes and their use as building‐block for *bay*‐extended nanographenes which opens the untapped potential of terrylene's chemistry and photophysics. Therefore, we developed a facile synthetic route for fully *peri*‐alkylated terrylene derivatives as solution‐processable functional materials motivated by their potential applications in optoelectronics, spectroscopy, singlet fission, organic field‐effect transistors (OFETs), organic light emitting diodes (OLEDs), pigments, dyes, and organic photovoltaics (OPVs).

## Results and Discussion

**Scheme 1:**
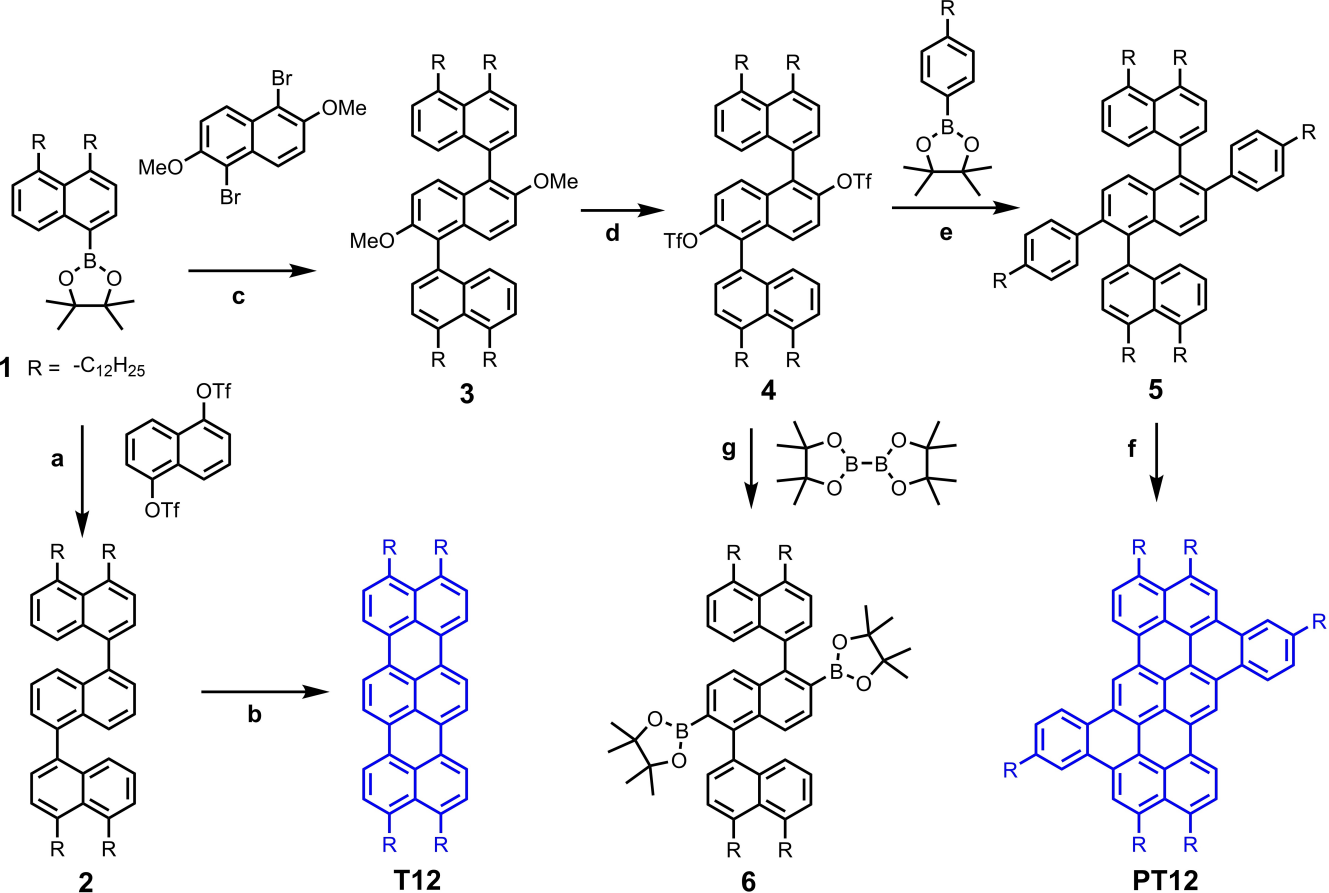
a) K_3_PO_4_, dry toluene, SPhos, Pd(dppf)Cl_2_, 100 °C, 24 h, 83 %; b) DCM, FeCl_3_, nitromethane, 0 °C to rt, 3 h, 44 %; c) K_2_CO_3_, Pd_2_(dba)_3_, XPhos, toluene, water, ethanol, 80 °C, 40 %; d) BBr_3_, DCM, 0 °C to rt, overnight, followed by trifluoromethanesulfonic anhydride, pyridine, DCM, 0 °C to rt, overnight, 85 %; e) K_3_PO_4_, dry toluene, SPhos, Pd(dppf)Cl_2_, 100 °C, 24 h, 73 %; f) DCM, FeCl_3_, nitromethane, 0 °C to rt, 3 h, 55 %; g) KOAc, Pd(dppf)Cl_2_, dioxane, 80 °C, 24 h, 74.4 %.

To realize the concept of *peri*‐alkylated terrylene, the synthesis requires appropriate coupling methods and building‐blocks (Scheme 1). Inspired by the seminal work^[16]^ in 1990’s, we have developed a *peri*‐alkylated naphthylboronic acid pinacol ester, 2‐(4,5‐didodecylnaphthalen‐1‐yl)‐4,4,5,5‐tetramethyl‐1,3,2‐dioxaborolane (**1**) to couple with 1,5‐dihaloginated naphthalene derivatives through Suzuki coupling reactions. Primary building block compound **1** was synthesized (Scheme S1, SI) through Kumada type coupling reaction of 1,8‐dibromonaphthalene with dodecyl‐zinc bromide followed by selective bromination with NBS and then converted to boronic acid pinacol ester. Compound **1** was coupled with 1,5‐naphthalenediyl bis(trifluoromethanesulfonate) in a two‐fold Suzuki reaction using Pd(dppf)Cl_2_ as catalyst, Sphos as ligand and K_3_PO_4_ as base in toluene at 100 °C to get ternaphthalene derivative, 4,4′′,5,5′′‐tetradodecyl‐1,1′ : 5′,1′′‐ternaphthalene (**2**) in an excellent yield, 83 %. However, prior to these optimized conditions, we had tried variety of 1,5‐dihalo naphthalene derivatives and Pd catalysts, but it was very difficult to separate mono‐coupled derivatives from di‐coupled compounds due to negligible separation between them on silica gel based chromatography techniques such as thin layer chromatography (TLC) and column chromatography.

The *peri*‐dodecyl terrylene, 1,8,9,16‐tetradodecyl terrylene (**T12**) was prepared from **2** upon FeCl_3_ mediated Scholl reaction (Scheme 1). The cyclodehydrogenation of ternaphthalene **2** with FeCl_3_ (6 equivalents for one hydrogen to be removed) proceeded easily at room temperature, with 60 % yield of crude **T12**. However, as anticipated, there were some chlorinated products observed in MALDI‐TOF analysis of the crude mixture. Thus **T12** was purified through repeated precipitation from DCM solutions with methanol (×5 times) and was obtained in a pure form in 44 % yield. The filtrate contained the soluble mixture of terrylene **T12** and its chlorinated derivatives but not detectable amounts of five‐membered ring containing perylene derivatives. It was reported that non‐alkylated naphthalene‐perylene derivative, 3‐(naphthalen‐1‐yl)perylene upon treatment with FeCl_3_ for 24hr led to formation of benzo[4,5]indeno[1,2,3‐cd]perylene, a five‐membered containing perylene fused naphthalene in 46 % yield.^[21]^ However, in our case, major product was *peri*‐alkylated terrylene and this could be due to dodecyl groups at *peri*‐positions. This is fully in agreement with our recorded ^1^H‐NMR (Figure S**1**) at 129 °C in C_2_D_2_Cl_4_.

For the *bay*‐extended terrylene synthesis, we procured 1,5‐dibromo‐2,6‐dimethoxynaphthalene and linked with compound **1** to get 2′,6′‐dimethoxy‐4,4′′,5,5′′‐tetramethyl‐1,1′ : 5′,1′′‐ternaphthalene (**3**) through Pd‐catalyzed Suzuki coupling. Due to methoxy groups, two‐fold coupling reaction required optimization of reaction solvents mixtures, temperature, catalyst/ligand combination. Solvent mixture of toluene/water/ethanol (5 : 1 : 1 ratio) worked better than THF/water (5 : 1) with K_2_CO_3_ base and Pd_2_(dba)_3_ to obtain compound **3** in 40 % yield. The other major side product from this reaction was mono‐coupled compound. After the demethylation of **3** upon treatment with BBr_3_, dihydroxy ternaphthalene was obtained in a quantitative yield, which was subsequently converted into pure bis(triflate) ternaphthyl derivative (**4**) in moderate yield after purification on silica gel column chromatography. It is worth noting, compound **4** is one of the key building blocks for *bay*‐extension of *peri*‐alkylated naphthalene and is more emphasized in the outlook. The triflate groups are very useful as they behave like halides (pseudo‐halides) and readily participate in C−C coupling reactions. Therefore, 4‐dodecyl‐phenylboronic ester was used in the Suzuki coupling reaction with compound **4**. Triflate **4** and 4‐dodecyl‐phenylboronic ester cross‐coupled under Suzuki conditions to provide **5** in a moderate to high yields. During the optimization of coupling reaction conditions, we observed that increasing the reaction temperature (90 to 120 °C) and employing a strong base (from K_3_PO_4_ to K_2_CO_3_) provide a greater mono‐coupled product (up to 37 %) with lower amounts of the two‐fold coupled product (**5**). The SPhos ligand was added to improve the reaction‘s efficiency, allowing the synthesis of compound **5** in 73 % yield. Therefore, bis(triflate) derivative of ternaphthalene, **4** would be a potential coupling partner for Suzuki type coupling reactions. We have also demonstrated the potential of compound **4** by converting it into diboronic acid pinacol ester **6** in 74 % yield, another key partner in Suzuki polymerization reactions. This reaction was tested to see whether it is possible to obtain the boronic ester in high yields and hence to perform polymerization reaction in the future.

The final cyclodehydrogenation of compound **5** with FeCl_3_ (6 equiv. to one reactive hydrogen) proceeded effortlessly at room temperature within 3 hours of reaction time, yielding phenylene‐fused terrylene, **PT12** as dark purple color flakes. After repeated precipitation from DCM solutions with methanol (×5 times) yielded 53 % pure **PT12** which is highly soluble in regular organic solvents such as THF, chloroform, toluene acetone, acetonitrile. However due to aggregation or strong π–π interactions between semi‐discotic **PT12** molecules, room temperature ^1^H‐NMR was not fully resolved. High temperature ^1^H‐NMR revealed clear doublets and singlets (Figure S2). The filtrate and washings of DCM‐methanol solutions indicate partial chlorination of fully fused compound (evident from MALDI‐TOF analysis), but NMR analysis show no indication of five‐membered rings after FeCl_3_ mediated cyclodehydrogenation.

To investigate the intermolecular interaction and the alignment of the alkyl chains in the solid state, single crystals of compounds **2**, **4** and **5** were grown by slow vapor diffusion of methanol into DCM solutions. The solid‐state structure of compounds **2**, **4** and **5** were determined by single crystal X‐ray diffraction crystallography (see SI, Table S1). Single crystal of all three molecules ternaphthalenes possesses triclinic P‐1 space group (Figures S4–S6). The naphthalene units in ternaphthalene were almost 90° (dihedral angles) twisted, because of high steric hindrance in the structure. The intermolecular π–π distance averages for **4** is higher than **2** because of triflate groups on the central naphthalene unit. One of the two alky chains on the naphthalene units was bent due to the hydrogen repulsion of the other alkyl chain which was clearly evident in all three compounds. The molecule **5** did not show any intermolecular π–π interactions, due to the phenyl rings at the middle naphthalene unit of the ternaphthalene which prevent any stacking. Attempts to grow single crystals of **T12** and **PT12** by slow vapor diffusion of methanol into DCM solutions at room temperature or tetrachloroethane solution at high temperature were unsuccessful due to strong aggregations of these molecules.

As compounds **T12** and **PT12** have a high tendency to aggregate due to strong π‐π interactions, NMR measurements at room temperature were not successful. To overcome the aggregation in these compounds, ^1^H NMR spectra of compounds **T12** and **PT12** were measured at 129 °C. Proton NMR spectra of *peri*‐alkylated terrylene **T12** showed three peaks expected for highly symmetric structure. Spectra of **PT12** showed three singlets and four doublets (Figure S**2**) which was in agreement with the expected product. Thermogravimetric analysis (TGA) of compounds was measured up to 900 °C and compounds showed thermal stability up to 360 °C. Differential Scanning Calorimetry (DSC) experiments were carried out (Figure S**7**) to investigate the different phase transitions in **T12** and **PT12**. Three cycles of heating to 300 °C and cooling to 0 °C were carried out for both compounds. From the DSC graph of compound **T12**, there was a clear reversible phase transition ~150 °C and the molecule displayed a melting peak at 195 °C. The *peri*‐alkylated terrylene's melting point was independently verified on melting point apparatus and confirmed. The melting point for *peri*‐alkylated terrylene is far below the melting point found in the literature for tetra‐*ortho*‐*tert*butyl terrylene (369–370 °C). In case of **PT12**, two peaks were defined which indicate different phase transition. The first peak shows a phase transition, which happened at 115 °C. Moreover, this is a reversible phase transition (crystallization). The other peak at 39.17 °C also demonstrates a reversible phase transition point while cooling to 0 °C. Because DSC was only measured until 300 °C, the melting point of **PT12** was found to be 335 °C (±1), which was determined using a melting point apparatus.

**Figure 2:**
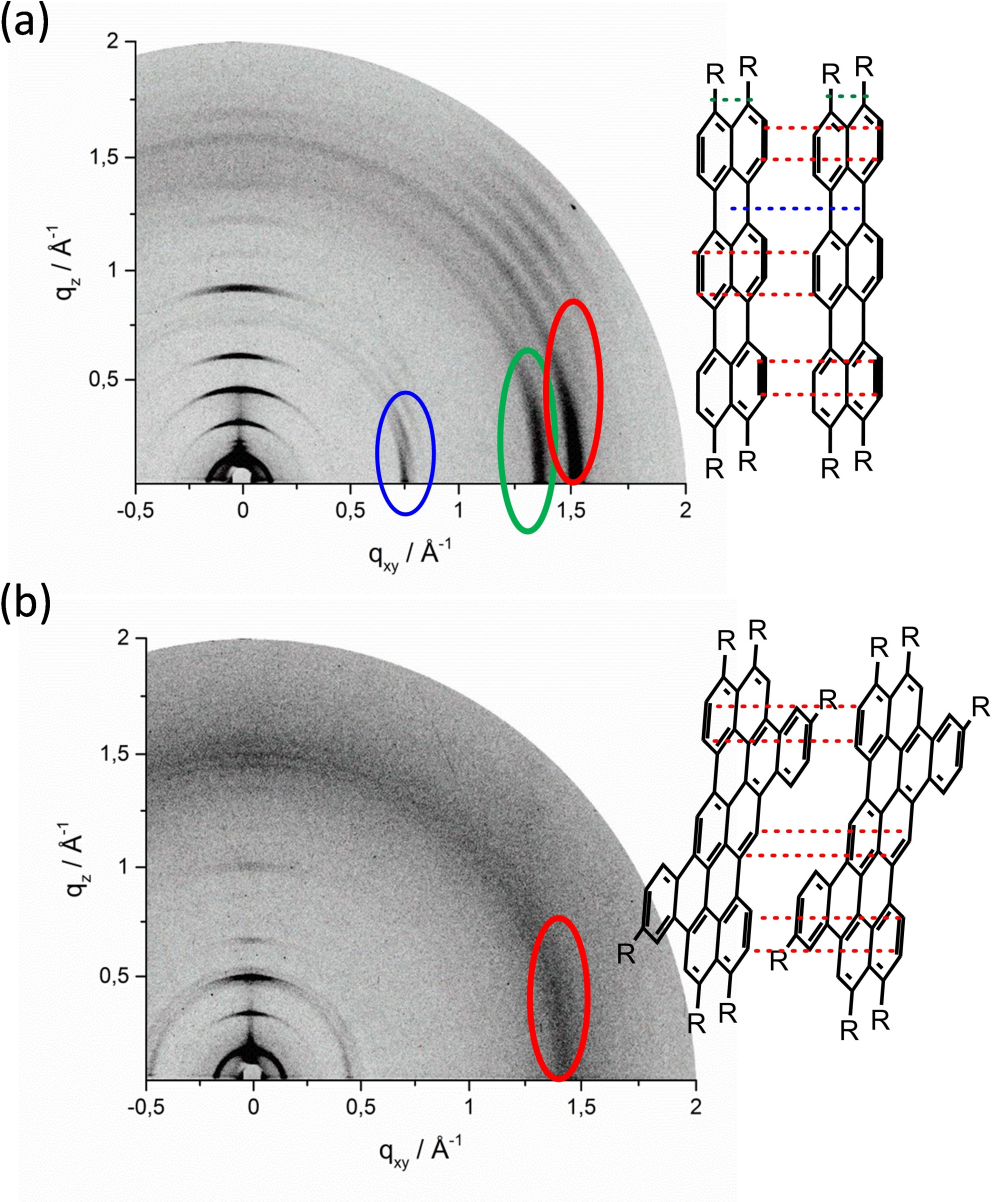
GIWAXS analysis of (a) **T12** and red inset highlights reflection assigned to possible π‐stacking interaction, blue inset indicates the width between two molecules and green inset displays distance between alkyl chains; (b) **PT12** and red inset highlights reflection assigned to π‐stacking interaction.

Grazing incidence wide‐angle X‐ray scattering (GIWAXS) measurements provide valuable information about the π‐ stacking interactions and the surface organization of the compounds in thin film. Because of the inverse nature of reciprocal‐space, these large values of q correspond to small distances and WAXS generically probes molecular length‐scales of compressed thin films. Compound **T12** has displayed high out‐of‐plane order (along q_z_ for q_xy_=0 Å^−1^) as shown by fourth‐order reflections (Figure 2a). The scattering intensities have displayed an edge‐on organization with an interlayer distance of 3.99 nm. Wide‐angle in‐plane reflection (red marking in Figure 2a) is attributed to π‐stacking interactions with 0.39 nm between conjugated backbones. In this edge‐on structure, π‐stacking is parallel to the surface, which could benefit charge carrier transport in FETs. The blue mark at q_xy_=0.75 Å^−1^ indicates the molecule‘s 0.80 nm width. The reflection at q_xy_=1.35 Å^−1^ indicates high order of alkyl chains with a distance of 0.46 nm highlighted in green. Compound **PT12** was well‐organized and long‐term order is diffused (Figure 2b). Compound **PT12** has a shorter interlayer distance of 3.68 nm and a larger π‐stacking distance of 0.42 nm compared to **T12**, indicating that π–π interactions in **T12** are stronger due to the linear alignment of the molecule. The fused benzene rings disrupt π‐stacking in compound **PT12**. Discotic molecules produce columnar packing like HBC (hexabenzocoronene), hence **T12** and **PT12** are likely to behave similarly.

**Figure 3:**
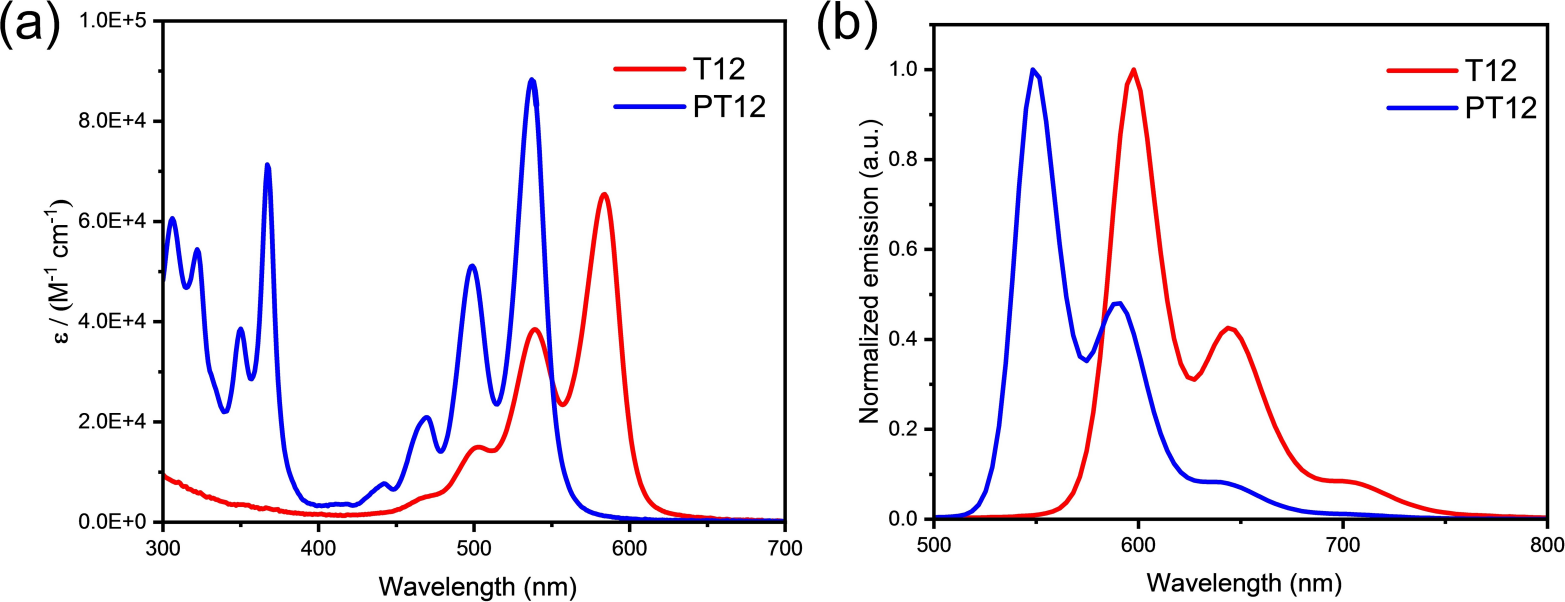
(a) Absorption spectra of *peri*‐alkylated terrylene **T12** (red) and phenylene‐fused terrylene **PT12** (blue) recorded in degassed toluene. (b) Fluorescence spectra of **T12** (λ_exc_=584, red) and phenylene‐fused terrylene **PT12** (λ_exc_=537, blue) in degassed toluene at room temperature.

Opto‐electronic properties of *peri*‐alkylated terrylene compounds **T12** and **PT12** were recorded in degassed toluene solutions and both absorption and fluorescence spectra were measured (Figure 3). Both compounds displayed typical rylene vibronic fine structure in the absorption spectra and mirror image of fluorescence spectra. The *peri*‐alkylated terrylene **T12** absorbs strongly at 502, 539, and 584 nm (λ_max_) and displays almost 25 nm red‐shift when compared to unsubstituted terrylene^[21]^ or *ortho*‐alkylated terrylene^[16]^ and 50 nm in contrast to *bay*‐alkylated terrylene.^[17]^ Alkyl chains at *peri*‐positions of terrylene did not affect the planarity of molecules and causes no out‐of‐plane distortions. In addition, alkyl chains at *peri*‐position improve the overlap of the π‐orbitals thus diminish the highest occupied molecular orbital (HOMO) ‐ lowest unoccupied molecular orbital (LUMO) gap and affecting the energy levels. **PT12** displayed strong longest wavelength band with rylene's characteristic vibronic structure and four peaks at 442, 470, 500, and 536 nm (λ_max_) and 48 nm blue shifted from λ_max_ of **T12**. The *bay*‐extension of terrylene core in the case of phenylene‐fused compound **PT12** lead to increases the HOMO‐LOMO gap, shifting absorption spectra hypsochromically. The molecular extinction coefficient of *peri*‐alkylated terrylene **T12** (65500 L mol^−1^cm^−1^ at 584 nm) slightly increases compared to the literature known terrylene (65000 L mol^−1^ cm^−1^ at 560 nm) but **P12** displayed very strong molecular extinction coefficient of 88700 L mol^−1^cm^−1^ at 536 nm.

Similar trends are seen in fluorescence spectra. The emission maxima (548 nm) for **PT12** were blue shifted from emission maxima of **T12** similar to absorption λ_max_. **PT12** fluorescence spectra have a 13 nm Stokes shift, while **T12** had 11 nm, and this again reflects the planar molecular structures and absence of any out‐of‐plane distortions in the structures. Surprisingly, **PT12** has displayed much higher fluorescence quantum yield Φ_F_ (90 %) than **T12** (72 %). The fluorescence quantum yield of **T12** slightly increased compared to *ortho*‐tert‐butylated terrylene (Φ_F_ 70 %)^[16]^ this might be due to lengthy alkyl chains at *peri*‐positions of terrylene.

**Figure 4:**
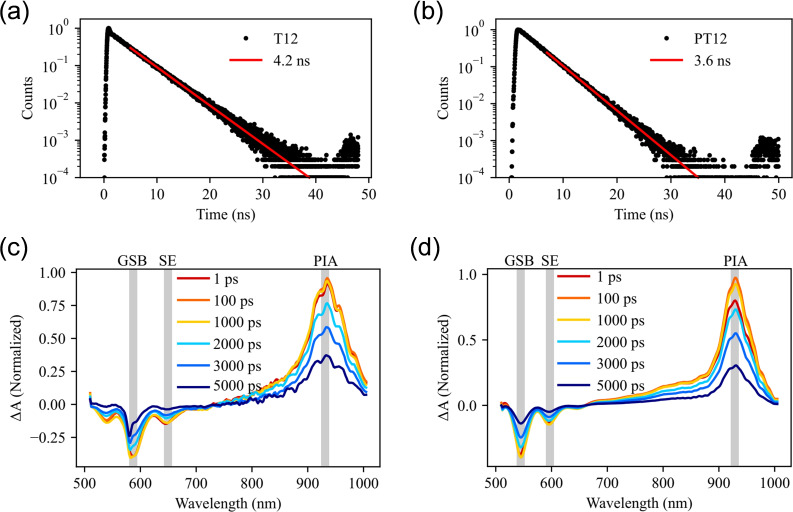
Time‐correlated single photon counting measurements of (a) **T12** and (b) **PT12**. Transient absorbance spectra of (c) **T12** and (d) **PT12**.

Time‐correlated single photon counting (TCSPC) studies of *peri*‐alkylated terrylene, **T12** showed a single‐exponential decay constant (Figure 4 a&b) in good agreement with previous photophysical studies of terrylene derivatives[^18^, ^41^, ^43^] (τ=4.2±0.2 ns), whereas **PT12** demonstrated a slightly faster decay constant (τ=3.6±0.3 ns). Transient absorption (TA) spectroscopy revealed similar excited state dynamics for both **T12** and **PT12** upon excitation at 584 nm and 537 nm, respectively. TA time slices showing photoinduced absorption (PIA) assigned to the singlet exciton state S_1_, stimulated emission (SE) and ground state bleach (GSB) features are plotted in Figure 4c and **4 d**. The kinetics of these features are fitted to the same nanosecond time constant parameters from TCSPC (see SI 5). Mirroring of the PIA kinetics with SE and GSB features (Figure S8) suggests minor intersystem crossing to form triplet excitons, and leads to relatively high fluorescence quantum yields in **T12** and **PT12**. Phenylene‐fused compound, **PT12** demonstrated a higher Φ_F_ of 0.90 compared to 0.72 for **T12**. From this information we determined the radiative and non‐radiative rate ($k_{r}$ and $k_{nr}$ ) using equations:

$$k_{r}=\frac{\Phi_{F}}{\tau }\;\mathrm{and}\;\Phi_{F}=\frac{k_{r}}{k_{r}+k_{nr}}$$

A summary of the photophysical properties derived are given in table 1. A similar $k_{r}$ is seen in both molecules, however the $k_{nr}$ for **PT12** is significantly lower than that of terrylene **T12** and follows the energy gap law trend for faster S_1_‐S_0_ internal conversion with lower energy S_1_.

**Table 1:** Summary of photophysical properties of synthesized compound **T12** and **PT12** and comparison with reported *ortho*‐alkylated terrylene.

Property measured	T12	PT12	ortho‐^t^bu terrylene^[16]^
Absorption λ_max (nm)_	584	537	560
Emission λ_max (nm)_	597	548	573
*ϵ* (L. mol^−1^.cm^−1^)	65500	88700	65000
$\Phi_{F}$	0.72	0.90	0.70
τ	4.2±0.2 ns	3.6±0.3 ns	3.8 ns
$k_{r}$	1.71±0.08×10^8^ s^−1^	2.50±0.14×10^8^ s^−1^	–
$k_{nr}$	6.67±0.32×10^7^ s^−1^	2.77±0.15×10^7^ s^−1^	–

DFT calculations are carried out using the B3LYP/6‐31G* functional on model systems analogues to synthesized molecules **T12** and **PT12** but replacing dodecyl chains with methyl groups and results are summarized (see SI, section 6) along with experimental results. *Peri*‐alkylated molecule was found to be completely planar whereas phenylene‐fused molecule had slightly twisted at extended positions (Figure S10). The HOMO, LUMO and energy levels of **PT12** and **T12** are following a similar trend that was predicted for rylenes. Rylenes show a unique progression of electronic structures that fundamentally determine all of their interesting photophysical properties. Theoretical calculations in the 1985 predicted that polyrylene would be better regarded as a π‐conjugated structure containing two interacting *cis*‐polyacetylene chains (Figure 1).^[4]^ In the case of terrylene model compounds, the coefficients for the (HOMO) and the (LUMO) are antisymmetric with respect to the mirror plane parallel to the molecules long axis (Figure S11). As a result, the coefficients on the carbon atoms located on that mirror plane vanish. Thus, contributions to the HOMO and LUMO only come from the atoms on the periphery of the long axis. Theoretical calculations also predict^[36]^ that the longest wavelength absorption band of a terrylene molecule will be mainly due to HOMO→LUMO excitation, which is polarized toward the long molecular axis. With extension of the π‐conjugation along the molecular long axis, the HOMO level rises; on the other hand, if *bay*‐extended, both the HOMO and LUMO levels are affected leading to observed hypochromic shift (Figure S12).

## Conclusions

A novel synthesis route was established to make highly soluble *peri*‐alkylated terrylene and extend the π‐conjugation at *bay*‐regions. We demonstrated synthesis of model molecules such as tetra *peri*‐dodecyl terrylene, **T12** and it's di‐phenylene‐fused at the *bay*‐positions of terrylene **PT12**. We have also synthesized two key building blocks (**4**, **6**) which would be very helpful to extend the edges of terrylene. The structural and photo physical properties of these two model compounds were evaluated by NMR, UV‐vis spectroscopy, DSC, GIWAXS and DFT calculations. High temperature NMR spectra unambiguously confirm the absence of any five‐membered ring containing side products. **PT12** showed remarkably high fluorescence quantum yield (0.90), due to π‐extension at *bay*‐positions which was also supported by TA spectroscopy, almost approaching the quantum yield of perylene (0.94). These key building blocks and model compounds can play essential role for *bay*‐extended polymeric compounds, which could serve as a starting material to multi‐edge graphene nanoribbons and PAHs. These nanoribbons or polymers can reveal unique structures, leading towards “chiral edges” and could play a promising role in charge transport studies and other applications.

## Supporting Information

Experimental methods including synthetic details and characterization such as ^1^H and ^13^C NMR, HR‐MS, single crystal XRD analysis (Figure S3–S6) including CCDC numbers, from 1519199 to 1519202, DSC (Figure S7), TA spectroscopy excited state dynamics (Figure S8), photoluminescence quantum yield (Figure S9) and DFT calculations (Figure S10–11) are given in the supporting information. The authors have cited additional references within the Supporting Information (Ref. [44–45]).

## Conflict of interests

The authors declare no conflict of interest.

1

## Supporting information

As a service to our authors and readers, this journal provides supporting information supplied by the authors. Such materials are peer reviewed and may be re‐organized for online delivery, but are not copy‐edited or typeset. Technical support issues arising from supporting information (other than missing files) should be addressed to the authors.

Supporting Information

## Data Availability

The data that support the findings of this study are available in the supplementary material of this article.
