# Supplementary material for: Peri‐Alkylated Terrylenes and Ternaphthalenes Building‐Blocks Towards Multi‐Edge Nanographenes
Source: Chemistry. 2024 Jun 3;30(36):e202401462. doi: 10.1002/chem.202401462 (PMC11497246; doi:10.1002/chem.202401462)
Supplement: Supplementary file 1 — Supporting Information [file CHEM-30-e202401462-s001.pdf]

Chemistry–A European Journal

Supporting Information

Peri-Alkylated Terrylenes and Ternaphthalenes Building-Blocks Towards Multi-Edge Nanographenes

Vikas Sharma, Hassan Khan, Michael Walker, Hamid Ahmad, Anmol Thanai, Tomasz Marszalek, Dieter Schollmeyer, Martin Baumgarten, Emrys W. Evans, and Ashok Keerthi*

Contents:

1. Methods and experimental details page 2

2. Synthesis page 2

3. Single crystal analysis and crystallographic data page 15

4. Differential scanning calorimetry page 24

5. Transient absorption and photoluminescence spectroscopy page 24

6. Density functional theory calculations page 26

7. ^{1}H-NMR, ^{13}C-NMR spectra of synthesized compounds page 28

8. MALDI-TOF analysis of synthesized compounds page 39

9. References page 42

Methods and experimental details

All reagents were purchased from commercial sources and used without further purification unless otherwise stated. Thin layer chromatography was carried out using pre-coated aluminum sheets with silica gel 60 F254 (Merck). Column chromatography was performed using Merck silica gel (60 Å, 230−400 mesh). ^{1}H-NMR and ^{13}C-NMR spectra were recorded in the listed deuterated solvents on a Bruker 400, and 500 MHz spectrometers (s – singlet, d – doublet, dd – doublet of doublet, t – triplet, b – broad, m – multiplet). Matrix-assisted laser desorption/ionization time-of-flight mass spectrometry (MALDI-TOF-MS) was performed using a Shimadzu Biotech AXIMA Confidence MALDI mass spectrometer in linear (positive) mode. Solution of 50 µL synthesized compounds (1 mg mL^{-1} in THF) was mixed with 50 µL of a 10 mg mL^{-1} solution of the matrix (dithranol or tetracyanoquinodimethane) in THF. A drop of this solution was spotted onto a MALDI plate which had been pre-spotted with sodium iodide in THF (10 mg mL^{-1}). UV-Vis absorption spectra and optical densities were measured using a Varian Cary 5000 UV-Vis-NIR spectrophotometer, while fluorescence emission spectra were gathered using a Varian Cary Eclipse fluorescence spectrophotometer at 298 K. The X-ray crystallographic data for the molecules were collected on a STOE IPDS 2T diffractometer using Cu-Kα IµS source.

2. Synthesis

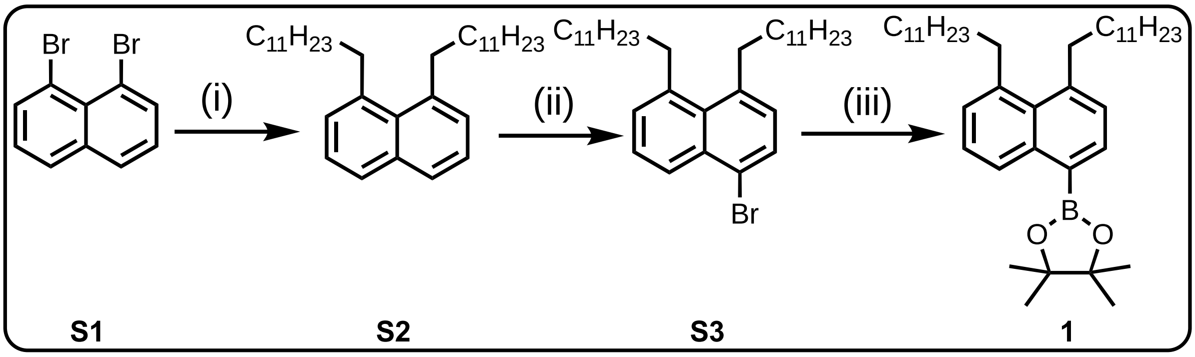

Scheme S1. (i) $C_{12}H_{25}ZnBr$, Pd(dppf)Cl_2, DMA, 130 ^{o}C, 2d, yield 81 %; (ii) 1,8-didodecylnaphthalene, NBS, DMF, 35 ^{o}C, overnight, yield 85 %; (iii) 1-bromo-4,5-didodecylnaphthalene, $B_2(Pin)_2$, KOAc, dry dioxane, Pd(dppf)Cl_2, 80 ^{o}C, 18 h, yield 88 %.

We have optimized the Kumada type cross-coupling reaction condition to convert cleanly the commercially available 1,8-dibromonaphtahlene (**S1**) into 1,8-didodecylnaphthalene (**S2**). We

began our studies by examining the cross-coupling using 1,8-dibromonaphtahlene as an electrophilic component and *n*-dodecyl magnesium chloride as a nucleophile in the presence of Ni-based catalysts such as Ni (dppp)Cl_2 and Ni(dppe)Cl_2 systems. But at the end a mixture of mono- and didodecyl naphthalene was obtained, which was very difficult to separate because of negligible separation between them on TLC. After extensive optimization, we found that the desired cross-coupling product could be obtained in 81% yield using *n*-dodecyl Zinc chloride as a nucleophile in the presence of Pd(dppf)Cl_2. Further the selective monobromination at *peri*-position (**S3**) was achieved by using NBS in DMF with excellent yield. Then brominated product was easily converted into the boronic acid (**1**) through Miyaura-borylation in 88 % yield.

1,8-Didodecylnaphthalene (S2)

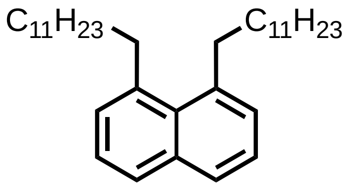

Dry N,N-dimethylacetamide (40 mL) was added to zinc (15.74 g, 240.74 mmol) and iodine (1.68 g, 6.62 mmol) in a schlenk tube, and stirred at room temperature until the purple colour of iodine disappeared. 1-Dodecyl bromide (10.00 g, 40.12 mmol) was then added to the mixture and stirred at 110 °C for 24 h to generate 1-dodecylzinc bromide. The solution of 1-dodecylzinc bromide was added to a mixture of 1,8-dibromonaphthalene (3.00 g, 10.88 mmol) and dichloro[1,1'- bis(diphenylphosphino)-ferrocene]palladium(II) (767 mg, 1.049 mol) in a 100-mL Schlenk flask. The reaction mixture was stirred at 120 °C for 15 h, and then quenched by hydrochloric acid (2 M, 30 mL). The aqueous layer was extracted three times with dichloromethane. The combined organic layers were washed three times with water, dried over magnesium sulphate, and evaporated. The crude product was purified by column chromatography with Hexane as eluent yielding a colorless oil (4.10 g, 8.82 mmol, yield 81 %). **MALDI-TOF**: m/z = 464.37, (100 %, M+) calc. 464.43. **^{1}H NMR** (500 MHz, $CDCl_3$) δ 7.72 (dd, *J* = 7.7, 1.8 Hz, 2H), 7.47 – 7.29 (m, 4H), 3.33 – 3.02 (m, 4H), 1.80 – 1.59 (m, 4H), 1.46 (dd, *J* = 10.6, 5.0 Hz, 4H), 1.40 – 1.29 (b, 32H), 0.91 (t, *J* = 6.9 Hz, 6H). **^{13}C NMR** (126 MHz, $CDCl_3$) δ 139.88, 136.20, 131.03, 129.36, 128.46, 124.96, 37.52, 33.57, 32.10, 29.96, 29.86, 29.83, 29.82, 29.74, 29.54, 22.86, 14.29.

1-Bromo-4,5-didodecylnaphthalene (S3)

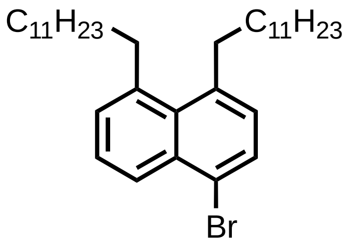

Under argon atmosphere didodecylnaphthalene (2.0 g, 4.30 mmol) was added in a flask and 200 ml of DMF was added. The flask was covered with aluminium foil. Every 5-10 min NBS (0.99 g, 5.59 mmol) was added in portion under room temperature. The reaction was stirred for further overnight and monitored by TLC. The reaction mixture was portion wise washed with 200-300 ml water each time and extracted with DCM. The collected DCM fractions were washed with Brine (NaCl). The organic phase was dried over $MgSO_4$ and solvent was removed under reduced pressure. The desired product was purified by column chromatography using flash silica gel in hexane to obtain a white solid (2.0 g, 3.678 mmol, yield 85 %). **Mp**: 45-46 °C. **MALDI-TOF**: m/z 542.29, 544.30, calculated 542.34, 544.34 **^{1}H NMR** (500 MHz, $CDCl_3$) δ 8.26 (dd, *J* = 8.4, 1.4 Hz, 1H), 7.66 (d, *J* = 7.7 Hz, 1H), 7.44 (dd, *J* = 8.4, 7.0 Hz, 1H), 7.37 (dd, *J* = 7.1, 1.4 Hz, 1H), 7.14 (d, *J* = 7.7 Hz, 1H), 3.14 – 3.05 (m, 4H), 1.63 – 1.55 (b, 4H), 1.44 – 1.36 (b, 4H), 1.33 – 1.20 (b, 32H), 0.89 (t, *J* = 6.9 Hz, 6H). **^{13}C NMR** (126 MHz, $CDCl_3$) δ 140.17, 139.88, 133.60, 132.23, 130.14, 129.23, 129.21, 127.21, 126.13, 122.31, 37.27, 37.24, 33.21, 33.02, 31.83, 29.63, 29.61, 29.58, 29.55, 29.52, 29.42, 29.26, 22.60, 14.03. **Elemental analysis $C_{34}H_{55}Br$ (%)**: calculated C, 75.11; H, 10.20; Br, 14.70; found C, 74.57; H, 11.26; Br, 14.17.

2-(4,5-Didodecylnaphthalen-1-yl)-4,4,5,5-tetramethyl-1,3,2-dioxaborolane (1)

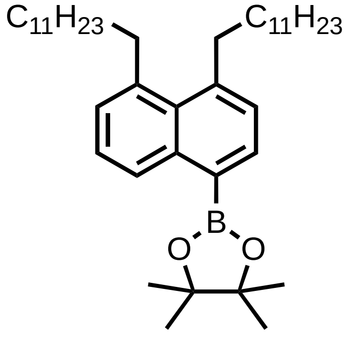

1-bromo-4,5-didodecylnaphthalene (2.20 g, 4.05 mmol), KOAc (794.21 mg, 8.09 mmol), bispinacoldiboron (1.54 g, 6.07 mmol) and the catalyst Pd(dppf)Cl_2 (296.07 mg, 0.045 mmol) were dissolved in 20 ml dry 1,4-dioxane in a 50 ml Schlenk-tube. After degassing with Argon for 30 min, the mixture was heated to 70 °C and stirred overnight. The reaction mixture was poured into water (200 ml) and extracted with DCM (3 x 75 ml) and dried over $MgSO_4$. After removing the solvent under reduced pressure (30°C). The crude product was purified by column chromatography with Hexane: DCM (10: 2) as eluent yielding a colorless oil (2.10 g, 3.55 mmol, yield 88 %). **MALDI-TOF**: m/z = 590.37 (calc. 590.52). **^{1}H NMR** (500 MHz, $CDCl_3$) δ 8.78 – 8.69 (m, 1H), 7.97 (d, *J* = 7.2 Hz, 1H), 7.43 – 7.39 (m, 1H), 7.33 (d, *J* = 6.2 Hz, 2H), 3.20 – 3.11 (m, 4H), 1.68 – 1.54 (b, 4H), 1.45 – 1.40 (b, 4H), 1.38 – 1.25 (b, 32H), 1.00 – 0.83 (m, 6H). **^{13}C NMR** (126 MHz, $CDCl_3$) δ 143.58, 139.84, 139.66, 135.01, 134.99, 130.91, 129.18, 128.68, 128.37, 125.39, 83.72, 38.10, 38.02, 33.44, 33.16, 32.09, 32.08, 29.82, 29.80, 29.53, 25.06, 22.86, 14.28, 14.27.

4,4'',5,5''-Tetradodecyl-1,1':5',1''-ternaphthalene (2)

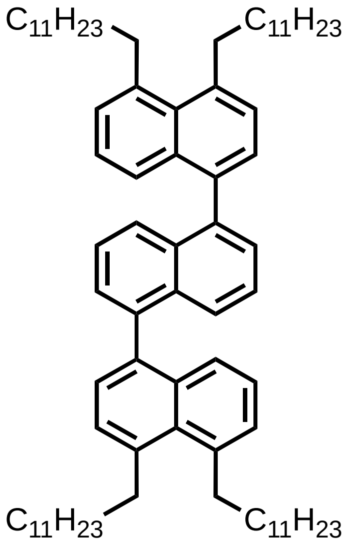

Under argon atmosphere naphthalene-1,5-diyl bis(trifluoromethanesulfonate) (150 mg, 0.353 mmole), compound **1** (626 mg, 1.06 mmole), K_3P0_4 (1.4 g), were dissolved in dry toluene (12 ml). After degassing with nitrogen for 30 minutes, the catalyst Pd(dppf)Cl_2 (39 mg, 0.053 mmole) and sphos (22 mg, 0.053 mmole) was added and further degassing with nitrogen for 30 min, the mixture was heated at 100 °C for 24 h. After passing the reaction mixture through celite the solvent was evaporated and the crude product was purified by flash column chromatography using Hexane: DCM (10: 1) as eluent to obtain a white solid (310 mg, 0.294 mmol, yield 83.33 %). **Mp**: 83-84 °C. **MALDI-TOF**: m/z = 1052.75 and 1053.75 (calc. =

1052.91 and 1053.91). **¹H NMR** (500 MHz, $CDCl_3$) δ 7.51 – 7.42 (m, 8H), 7.39 (d, *J* = 7.1 Hz, 2H), 7.37 – 7.30 (m, 4H), 7.23 (dd, *J* = 8.4, 6.9 Hz, 1H), 7.17 (dd, *J* = 8.4, 7.0 Hz, 1H), 3.38 – 3.06 (m, 8H), 1.83 – 1.70 (b, 8H), 1.56 – 1.47 (b, 8H), 1.44 – 1.25 (b, 64H), 0.92 (t, *J* = 6.9 Hz, 12H).**¹³C NMR** (126 MHz, $CDCl_3$) δ 140.03, 140.01, 139.95, 139.70, 138.33, 135.48, 133.22, 131.24, 131.21, 129.36, 129.32, 128.81, 127.85, 127.37, 127.34, 127.01, 126.97, 126.60, 125.42, 125.40, 125.00, 124.94, 37.82, 37.78, 33.73, 33.66, 32.11, 29.89, 29.87, 29.86, 29.80, 29.55, 22.87, 14.30. **Elemental analysis $C_{78}H_{116}$ (%)**: calculated C, 88.90; H, 11.10; found C, 88.82; H, 11.02.

1,8,9,16-Tetradodecyltribenzo[de,kl,rst]pentaphene (T12)

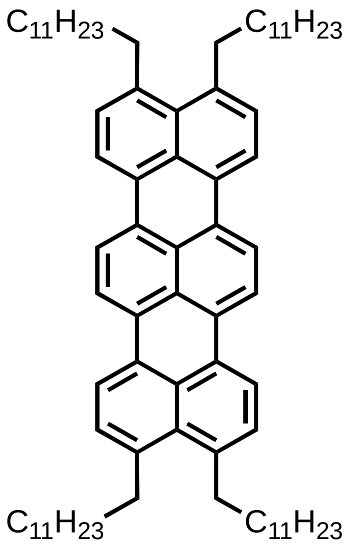

4,4'',5,5''-tetradodecyl-1,1':5',1''-ternaphthalene **2** (80.00 mg, 0.076 mmol) was dissolved in 20 ml DCM and degassed for 1 hour. After also degassing $FeCl_3$ solution 1.00 g in 5 ml nitromethane, $FeCl_3$ (295.52 mg, 1.82 mmol) was added dropwise at 0 °C, and the reaction mixture was stirred for further 3 hours at room temperature. The reaction was quenched by addition of 30 mL methanol which leads to precipitation of the desired product which was filtered over a membrane filter (0.25 µm PTFE) to obtain black powder. Repeated precipitation and filtration (5 × times) increases the purity (35 mg, 0.033 mmol, yield 43.96 %). **Mp**: 197-198 °C. **MALDI-TOF**: m/z = 1048.71 (calc. = 1048.88). **¹H NMR** (500 MHz, $C_2D_2Cl_4$) δ 8.22 (s, 4H), 8.17 (s, 4H), 7.44 (d, *J* = 10 Hz, 4H), 3.25 (b, 8H), 1.79 (b, 8H), 1.54 (m, 8H), 1.39 (m, 64H), 1.09 - 0.96 (m, 12H). **Elemental analysis $C_{78}H_{112}$ (%)**: calculated C, 89.25; H, 10.75; found C, 88.60; H, 9.58.

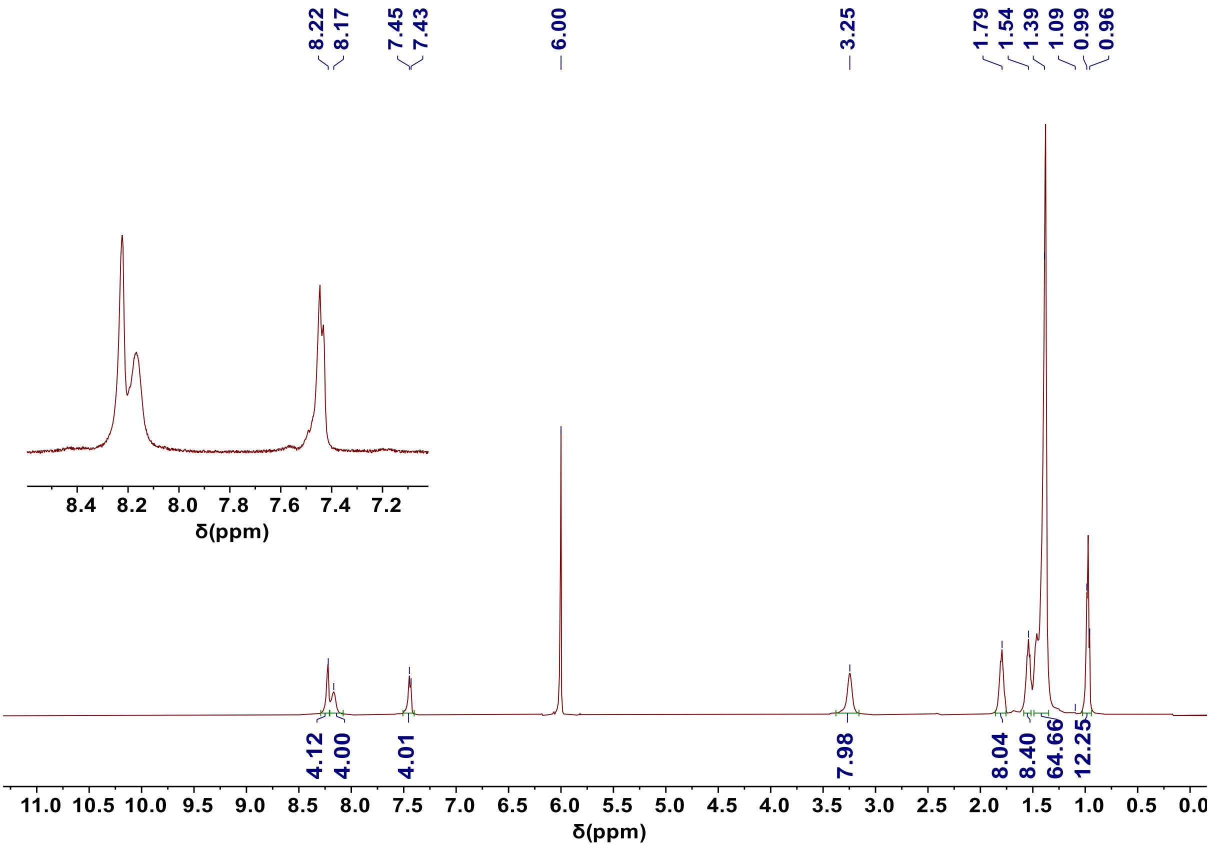

Figure S1. ^{1}H-NMR spectra of **T12** in 1,1,2,2-tetrachloro ethane-D2 at 130 °C.

4,4'',5,5''-Tetradodecyl-2',6'-dimethoxy-1,1':5',1''-ternaphthalene (3)

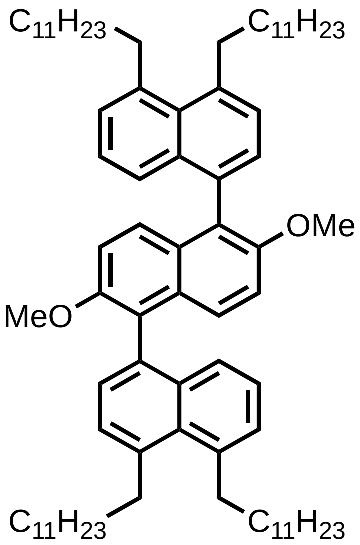

2,6-dimethoxy-1,5-dibromonaphatalene (132.00 mg, 0.38 mmol), 2-(4,5-didodecylnaphthalen-1-yl)-4,4,5,5-tetramethyl-1,3,2-dioxaborolane (498 mg, 0.84 mmol) and K_2CO_3 (793 mg, 5.74 mmol) were dissolved in 10 ml dry Toluene and 2 ml water and 2 ml ethanol. The reaction mixture was degassed by nitrogen bubbling for 30 minutes at room temperature, and then

$Pd_2(dba)_3$ (35 mg, 0.038 mmol) and XPhos (55mg, 0.114 mmol) was added and again degassed by nitrogen bubbling for more 30 min. After stirring at 110 °C for 18 hours under nitrogen atmosphere, the reaction mixture was allowed to cool to room temperature. The reaction mixture was poured into water (50 ml) and extracted with DCM (3 x 30 ml) and dried over $MgSO_4$. After removal of the solvents under reduced pressure, the residual oil was purified by column chromatography using flash silica gel with Hexane: DCM (10: 0.5) as eluent to yield a white solid (166 mg, 0.149 mmol, yield 39 %). **Mp**: 85-87 °C. **MALDI-TOF**: m/z = 1112.87 and 1113.87 (calc. = 1112.93 and 1113.93). **^{1}H NMR** (500 MHz, $CDCl_3$) δ 7.48 (t, *J* = 6.9 Hz, 2H), 7.41 – 7.36 (m, 2H), 7.35 – 7.30 (m, 4H), 7.24 – 7.13 (m, 6H), 3.64 (s, 6H), 3.37 – 3.09 (m, 8H), 1.84 – 1.71 (b, 8H), 1.59 – 1.47 (b, 8H), 1.44 – 1.22 (b, 64H), 0.90 (t, *J* = 6.8 Hz, 12H). **^{13}C NMR** (126 MHz, $CDCl_3$) δ 153.04, 140.03, 139.46, 135.47, 134.32, 131.49, 130.11, 129.26, 129.03, 127.82, 127.13, 126.44, 124.90, 124.52, 124.49, 114.79, 57.01, 37.80, 37.74, 33.75, 33.65, 32.10, 30.13, 30.11, 29.90, 29.87, 29.85, 29.82, 29.80, 29.55, 22.87, 14.30.**Elemental analysis $C_{80}H_{120}O_2$ (%)**: calculated C, 86.27; H, 10.86; O, 2.87; found C, 86.26; H, 11.61; O, 2.13.

4,4'',5,5''-Tetradodecyl-[1,1':5',1''-ternaphthalene]-2',6'-diol (4')

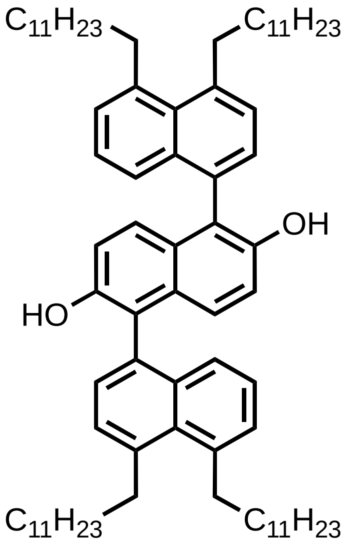

Under argon atmosphere 4,4'',5,5''-tetradodecyl-2',6'-dimethoxy-1,1':5',1''-ternaphthalene **3** (400.00 mg, 0.359 mmol) was dissolved in dry DCM (3 ml) and cooled to 0 °C and BBr_3 (1.51 mmol, 0.145 ml) was added to the reaction mixture. The reaction mixture was left stirring overnight at room temperature, quenched with water (200 ml), extracted with Dichloromethane (DCM) (3 x 75 ml) and dried over $MgSO_4$. After removal of the solvents under reduced pressure, the residual oil was purified by column chromatography using Hexane: EA (10: 0.5)

to obtain white very vicious oil in quantitative yield (390 mg, 0.359 mmol). **MALDI-TOF**: m/z = 1083.11 and 1084.11 (calc. = 1084.90 and 1085.90). **^{1}H NMR** (500 MHz, $CDCl_3$) δ 7.51 (t, *J* = 6.9 Hz, 2H), 7.48 – 7.45 (m, 2H), 7.43 – 7.35 (m, 4H), 7.30 – 7.27 (m, 1H), 7.23 (dd, *J* = 8.4, 7.0 Hz, 1H), 7.13 (dd, *J* = 9.1, 4.3 Hz, 2H), 7.07 (dd, *J* = 9.1, 1.4 Hz, 2H), 4.72 (s, 2H), 3.43 – 3.15 (b, 8H), 1.76 (b, 8H), 1.56 – 1.46 (b, 8H), 1.42 – 1.20 (b, 64H), 0.89 (t, *J* = 6.8 Hz, 12H). **^{13}C NMR** (126 MHz, $CDCl_3$) δ 149.23, 141.35, 140.53, 140.51, 135.37, 135.35, 132.01, 130.11, 129.46, 129.45, 129.41, 129.15, 126.90, 125.97, 125.93, 120.49, 120.46, 117.81, 117.78, 37.82, 37.72, 33.76, 33.61, 30.11, 30.07, 29.88, 29.86, 29.84, 29.77, 29.54, 22.86, 14.29. **Elemental analysis $C_{78}H_{116}O_2$ (%)**: calculated C, 86.28; H, 10.77; O, 2.95; found C, 86.41; H, 10.37.

4,4'',5,5''-Tetradodecyl-[1,1':5',1''-ternaphthalene]-2',6'-diyl bis(trifluoromethanesulfonate) (4)

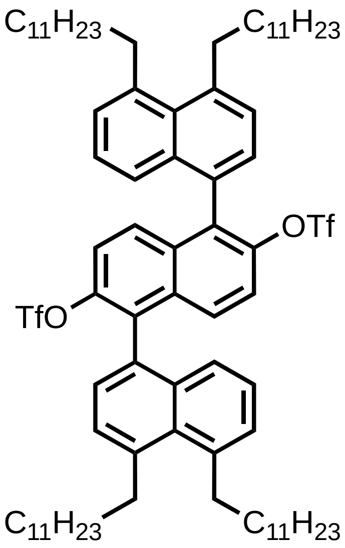

To a 50-mL round-bottom flask under nitrogen atmosphere, compound **4'** (500 mg, 0.46 mmol) and Pyridine (200 mg (0.15 ml), 1.84 mmol) were added and dissolved in DCM (15 mL). The reaction mixture was cooled to -10 °C and trifluoromethanesulfonic anhydride (519 mg (0.3 mL), 1.84 mmol) was added. The reaction mixture was stirred at rt for 12 hours under nitrogen atmosphere. The reaction was quenched with water and 1M HCl solution and extracted with DCM. The combined organic layers were washed with saturated aqueous $NaHCO_3$ solution and brine and then dried. After removal of the solvents under reduced pressure, the residual oil was purified by column chromatography using Hexane: DCM (10:3) to yield a yellowish white solid (550 mg, 0.407 mmol, yield 88.57 %). **Mp**: 59-60 °C. **MALDI-TOF**: m/z 1348.67 (100

%, M+), calculated 1348.79. **^{1}H NMR** (500 MHz, $CDCl_3$) δ 7.55 (dd, *J* = 9.3, 8.1 Hz, 2H), 7.49 (t, *J* = 7.0 Hz, 2H), 7.43 (d, *J* = 7.2 Hz, 2H), 7.39 – 7.32 (m, 4H), 7.24 – 7.17 (m, 3H), 7.12 (dd, *J* = 8.4, 1.4 Hz, 1H), 3.32 – 3.09 (b, 8H), 1.80 – 1.66 (b, 8H) 1.50 – 1.44 (b, 8H), 1.40 – 1.22 (b, 64H), 0.99 – 0.84 (m, 12H). **^{13}C NMR** (126 MHz, $CDCl_3$) δ 145.80, 141.84, 140.54, 140.49, 134.74, 133.49, 133.43, 132.57, 131.44, 129.76, 129.31, 129.28, 128.62, 125.74, 121.21, 37.90, 37.79, 33.50, 33.39, 32.10, 30.01, 29.96, 29.88, 29.85, 29.77, 29.55, 22.87, 14.29. **Elemental analysis $C_{80}H_{114}F_6O_6S_2$ (%)**: calculated C, 71.18; H, 8.51; F, 8.44; O, 7.11; S, 4.75; found C, 71.64; H, 8.83; S, 5.16.

2-(4-Dodecylphenyl)-4,4,5,5-tetramethyl-1,3,2-dioxaborolane

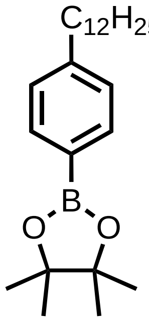

Under argon atmosphere 1-bromo-4-dodecylbenzene (3.00 g, 9.22 mmol), KOAc (1.81 g, 18.44 mmol), Bispinacoldiboron (4.68 g, 18.44 mmol) and the catalyst Pd(dppf)Cl_2 (1.01 g, 15% mol) were dissolved in 20 ml 1,4-dioxane in a 50 ml Schlenk-tube and was degassed with argon for 30 min. The mixture was heated to 85 °C and left stirring overnight. The reaction mixture was poured into water (200 ml) and extracted with DCM (3 x 75 ml) and dried over $MgSO_4$. After removing the solvent under reduced pressure (30°C) the crude product was purified by column chromatography using Hexane: DCM (10: 2) as eluent to obtain a colorless, vicious oil (2.80 g, 7.52 mmol, yield 81.54 %). **^{1}H NMR:** (500 MHz, $CDCl_3$) δ 7.74 (d, *J* = 8.0 Hz, 2H), 7.20 (d, *J* = 8.0 Hz, 2H), 2.65 – 2.58 (m, 2H), 1.62 (q, *J* = 5.9 Hz, 2H), 1.35 (s, 12H), 1.32 – 1.29 (b, 4H), 1.28 – 1.23 (b, 14H), 0.93-0.87 (m, 3H).**^{13}C NMR:** (126 MHz, $CDCl_3$) δ 146.58, 134.95, 128.04, 83.73, 36.34, 32.07, 31.50, 29.82, 29.81, 29.79, 29.74, 29.65, 29.51, 29.46, 25.00, 22.84, 14.27.

4,4'',5,5''-Tetradodecyl-2',6'-bis(4-dodecylphenyl)-1,1':5',1''-ternaphthalene (5)

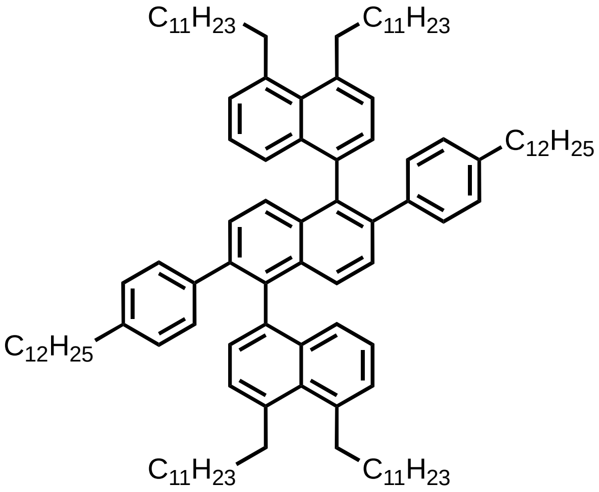

4,4'',5,5''-tetradodecyl-[1,1':5',1''-ternaphthalene]-2',6'-diyl bis(trifluoromethanesulfonate) **4** (300 mg, 0.22 mmol), 1-Boronicester-4-dodecylbenzene (248 mg, 0.66 mmol), potassium phosphate K_3PO_4 (933.98 mg, 4.40 mmol) were dissolved in 15 ml dry toluene. The reaction mixture was degassed by nitrogen bubbling for 30 minutes at room temperature, and then Pd(dppf)Cl_2 (24 mg, 0.033 mmol) and SPhos (13.5 mg, 0.033 mmol) was added and again degassed by nitrogen bubbling for more 30 min. Further reaction mixture was heated to 100 °C for 24 hours. The reaction mixture was poured into water (100 ml) and extracted with DCM (3 x 50 ml) and dried over $MgSO_4$. After removing the solvent under reduced pressure, the crude product was purified by flash column chromatography using Hexane: DCM (10:0.5) as eluent to obtain a white solid (250 mg, 0.162 mmol, yield 73.66 %). **Mp**: 78-80 °C. **MALDI-TOF**: m/z = 1542.05 (calc. = 1542.35). **^{1}H NMR** (500 MHz, $CDCl_3$) δ 7.54 (dd, *J* = 8.3, 1.5 Hz, 1H), 7.49 – 7.39 (m, 5H), 7.29 (ddd, *J* = 7.2, 5.7, 1.5 Hz, 2H), 7.27 – 7.21 (m, 3H), 7.21 – 7.16 (m, 2H), 7.13 (d, *J* = 7.2 Hz, 1H), 6.91 (dd, *J* = 8.2, 1.9 Hz, 4H), 6.77 (d, *J* = 8.0 Hz, 4H), 3.27 – 3.05 (b, 8H), 2.41 (t, *J* = 7.7 Hz, 4H), 1.88 – 1.61 (b, 8H), 1.47 – 1.14 (b, 112H), 0.93 – 0.84 (m, 18H). **^{13}C NMR** (126 MHz, $CDCl_3$) δ 140.70, 139.89, 139.36, 139.34, 139.18, 138.98, 136.92, 136.83, 136.35, 132.65, 130.96, 130.93, 129.09, 128.94, 128.80, 128.71, 128.64, 128.59, 127.45, 127.22, 127.11, 124.99, 124.94, 35.60, 33.33, 32.11, 32.09, 31.35, 30.02, 29.93, 29.90, 29.86, 29.83, 29.81, 29.78, 29.66, 29.56, 29.53, 29.41, 22.87, 22.85, 14.29, 14.27. **Elemental analysis $C_{114}H_{172}$ (%)**: calculated C, 88.76; H, 11.24; found C, 88.52; H, 11.48.

3,4,7,13,14,17-Hexadodecyldinaphtho[8,1,2-ijk:8',1',2'-vwx]pyranthrene (PT12)

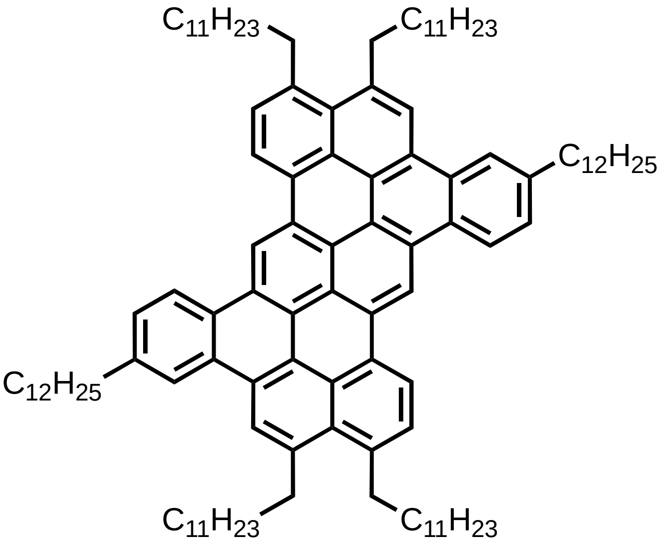

4,4'',5,5''-tetradodecyl-2',6'-bis(4-dodecylphenyl)-1,1':5',1''-ternaphthalene **5** (80.00 mg, 0.052 mmol) was dissolved in 20 ml DCM and degassed for 1 hour After also degassing $FeCl_3$ solution 1g in 5 ml nitromethane, $FeCl_3$ (403.75 mg, 2.489 mmol) was added dropwise, and the reaction mixture was stirred for further 3 h. The reaction was quenched by 20-30 ml methanol which leads to precipitation of the desired product which was filtered over a membrane filter to obtain black flakes. Repeated precipitation and filtration increase the purity (42 mg, 0.0273 mmol, yield 52.8 %). **MALDI-TOF**: m/z = 1534.13 and 1535.08 (calc. = 1534.28 and 1535.29). **^{1}H NMR** (500 MHz, $C_2D_2Cl_4$) δ 10.24 (s, 2H), 9.26 (d, *J* = 10 Hz, 2H), 9.12 (d, *J* = 10 Hz, 2H), 8.87 (s, 2H), 8.83 (s, 2H), 7.93 (d, *J* = 7.9 Hz, 2H), 7.88 (d, *J* = 7.9 Hz, 2H), 3.61 – 3.54 (m, 8H), 3.18 (m, 4H), 2.05 – 1.99 (m, 12H), 1.66 (m, 12H), 1.55 – 1.38 (b, 96H), 0.98 – 0.95 (m, 18H). **Elemental analysis $C_{114}H_{164}$ (%)**: calculated C, 89.23; H, 10.77; found C, 87.26; H, 9.88.

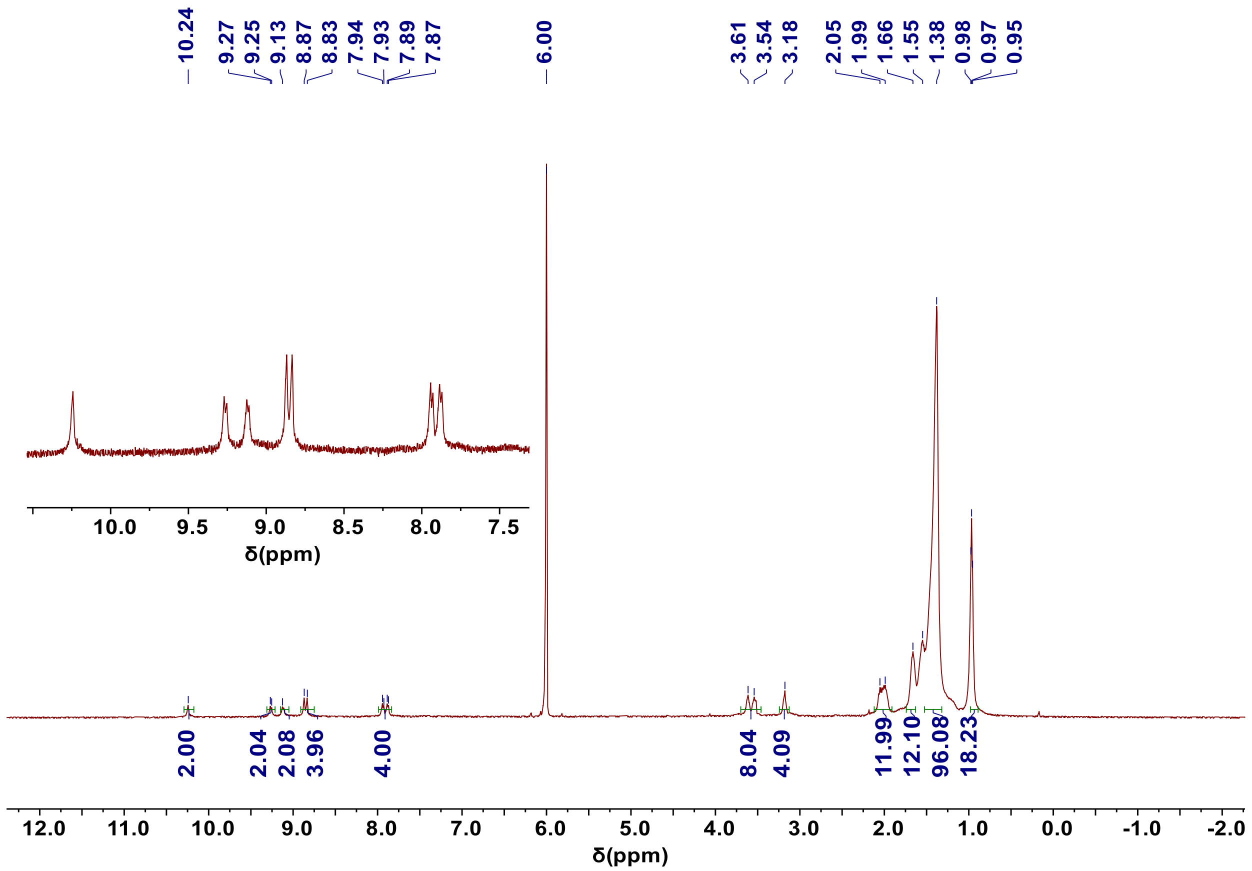

Figure S2. ^{1}H-NMR spectra of **PT12** in 1,1,2,2-tetrachloro ethane-D2 at 130 °C.

2,2'-(4,4'',5,5''-Tetradodecyl-[1,1':5',1''-ternaphthalene]-2',6'-diyl)bis(4,4,5,5-) tetramethyl-1,3,2-dioxaborolane (6)

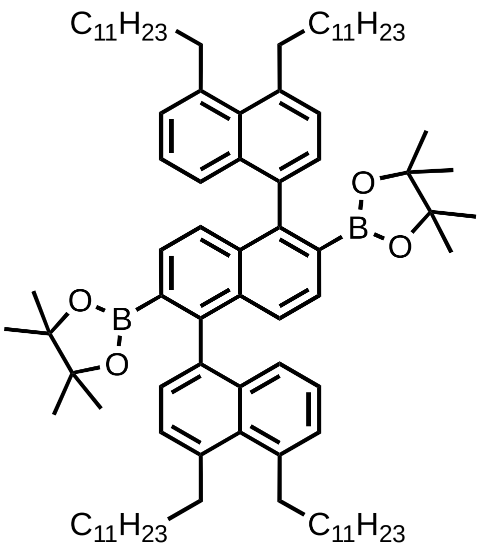

Under argon atmosphere 4,4",5,5"-tetradodecyl-[1,1':5',1"-ternaphthalene]-2',6'-diyl bis(trifluoromethanesulfonate) **4** (100 mg, 0.74 mmol) was dissolved in 1,4-Dioxane and degassed with Argon for 30 min. After the dropwise addition of pinacolborane (474.03 mg, 3.70 mmol) the catalyst Pd(dppf)Cl_2 was added, and the reaction mixture was refluxed overnight. The reaction mixture was poured into water, extracted with DCM and dried over $MgSO_4$. After removing the solvent under reduced pressure (30 °C) the crude product was purified by column chromatography using Hexane: DCM (10: 2) as eluent to obtain a colorless, vicious oil (72 mg, 0.055 mmol, yield 74.44 %). **MALDI-TOF**: m/z = 1305.28 and 1306.26 (calc. = 1305.08 and 1306.08). **^{1}H NMR** (300 MHz, CD_2Cl_2) δ 7.92 (d, *J* = 9.2 Hz, 1H), 7.56 (d, *J* = 8.5 Hz, 2H), 7.51 – 7.39 (m, 4H), 7.37 – 7.22 (m, 4H), 7.19 – 7.05 (m, 3H), 3.36 – 2.72 (m, 8H), 1.92 – 1.62 (b, 8H), 1.59 – 1.13 (m, 72H), 0.97 – 0.82 (m, 24H), 0.77 (s, 12H). **^{13}C NMR** (75 MHz, CD_2Cl_2) δ 146.23, 140.16, 139.72, 138.85, 136.62, 134.13, 132.40, 131.31, 129.90, 128.81, 127.88, 126.13, 124.84, 123.02, 83.57, 54.56, 54.20, 53.84, 53.48, 53.12, 38.09, 36.68, 34.12, 33.97, 32.38, 31.65, 30.25, 30.12, 30.06, 29.82, 24.46, 23.14, 14.32. **Elemental analysis $C_{90}H_{138}B_2O_4$ (%)**: calculated C, 82.79; H, 10.65; found C, 82.39; H, 10.98.

3. Single crystal analysis and crystallographic data

Table S1. CCDC reference numbers for single crystal data (crystallographic information files) of synthesized compounds.

IUPAC Chemical Name	Code in Publication		Unit Cell Parameters
1-bromo-4,5-didodecylnaphthalene (C34 H55 Br1)	**S3**	CCDC 1519199	a 4.9475(3)<br>b 79.883(6)<br>c 7.8592(5)<br>space group: Cc
4,4'',5,5''-tetradodecyl-1,1':5',1''-ternaphthalene (C78 H116)	**2**	CCDC 1519200	a 7.2208(7)<br>b 7.7187(9)<br>c 30.080(4)<br>space group: P-1
4,4'',5,5''-tetradodecyl-[1,1':5',1''-ternaphthalene]-2',6'-diyl bis(trifluoromethanesulfonate) (C80 H114 F6 O6 S2)	**4**	CCDC 1519201	a 6.9287(6)<br>b 9.4778(7)<br>c 30.345(3)<br>space group: P-1
4,4'',5,5''-tetradodecyl-2',6'-bis(4-dodecylphenyl)-1,1':5',1''-ternaphthalene (C114 H172)	**5**	CCDC 1519202	a 6.7005(7)<br>b 18.2040(18)<br>c 21.190(2)<br>space group: P-1

3.1. Crystal data for 1-bromo-4,5-didodecylnaphthalene (S3)

Molecular formula	$C_{34}H_{55}Br$
formula weight	543.69 $gmol^{-1}$
space group	C c (monoclinic)
Absorption	μ = 1.92 mm^{-1}
Transmission	T_{min} = 0.757, T_{max}= 0.981
Crystal size	0.02 x 0.1 x 0.24 mm^3 colorless plates
Lattice parameters	a = 4.9475(3) Å
	b = 79.883(6) Å ß = 90.491(5) °
	c = 7.8592(5) Å
Volume	V= 3106.0(4) $Å^3$
Z-Value	4
F(000)	1176.0
Temperature	-80°C
Density	$d_{x\text{-}ray}$ =1.163 gcm^{-3}
Scan type	ω scans
Scan collection	1°
Theta range for data collection	$2° \leq \theta \leq 68.8°$
Limiting indices	$-5 \leq h \leq 5$ $-94 \leq k \leq 94$ $-9 \leq l \leq 9$
total number of reflections	14539
Unique number of reflections	4882 (R_{int}=0.1651)
Observed number of reflections	3790 ($\|F\|/\sigma(F)$ > 4.0)
Structure solution	Programm: SIR-2004 (Direct Methods)
R-Values	wR2 = 0.2471 (R1 = 0.0817 for observed reflections, 0.1024 for all reflections)
Goodness of fit	S = 1.064
Max shift/Error	0.001 * e.s.d
Largest diff. Peak and hole	1.09, -1.06 $eÅ^{-3}$

3.2. Crystal data for 4,4'',5,5''-tetradodecyl-1,1':5',1''-ternaphthalene (2)

Molecular formula	$C_{78}H_{116}$	
formula weight	1053.70 $gmol^{-1}$	
space group	P -1 (triclinic)	
Absorption	μ = 0.059 mm^{-1}	
Crystal size	0.01 x 0.130 x 0.240 mm^3 colorless plates	
Lattice parameters	a = 7.2208(7)Å	α = 83.163(10)°
	b = 7.7187(9)Å	β = 82.983(9)°
	c = 30.080(4)Å	γ = 82.663(8)°
Volume	V = 1641.2(3)$Å^3$	
Z-Value	1	
F(000)	584.0	
Temperature	-143°C	
Density	$d_{x\text{-}ray}$ = 1.066 gcm^{-3}	
Scan type	ω scans	
Scan collection	1°	
Theta collection for data collection	$2° \leq \theta \leq 28°$	
Limiting indices	$-9 \leq h \leq 9$ $-10 \leq k \leq 10$ $-39 \leq l \leq 40$	
total number of reflections	15372	
Unique number of reflections	8044 (R_{int} = 0.1226)	
Observed number of reflections	2098 ($\|F\|/\sigma(F) > 4.0$)	
Structure solution	SIR-2004 (Direct methods)	
R-Values	wR2 = 0.2886 (R1 = 0.0884 for observed reflections, 0.2789 for all reflections)	
Fitgüte	S = 0.870	
Max shift/Error	0.001 * e.s.d	
Largest diff. Peak and hole	0.27, -0.33 $eÅ^{-3}$	
Remarks	Molecule is centrosymmetric	

3.3. Crystal data for 4,4'',5,5''-tetradodecyl-[1,1':5',1''-ternaphthalene]-2',6'-diylbis(trifluoromethanesulfonate) (4)

Molecular formula	$C_{80}H_{114}F_6O_6S_2$	
formula weight	1349.83 $gmol^{-1}$	
space group	P -1 (triclinic)	
Absorption	μ = 0.136 mm^{-1}	
Transmission	T_{min} = 0.9418, T_{max}= 0.9955	
Crystal size	0.020 x 0.060 x 0.960 mm^3 farblose Nadel	
Lattice parameters	a = 6.9287(6) Å	α = 90.461(6)°
	b = 9.4778(7) Å	β = 90.135(7)°
	c = 30.345(3) Å	γ = 109.315(6)°
Volume	V = 1880.5(3) $Å^3$	
Z-Value	1	
F(000)	728	
Temperature	-80°C	
Density	d_{x-ray} = 1.192 gcm^{-3}	
Scan type	ω scans	
Scan collection	1°	
Theta range for data collection	2° ≤ θ ≤ 28°	
Limiting indices	-9 ≤ h ≤ 9 -12 ≤ k ≤ 12 -39 ≤ l ≤ 40	
total number of reflections	18475	
Unique number of reflections	9246 (R_{int} = 0.0712)	
Observed number of reflections	4643 (|F|/σ(F) > 4.0)	
Structure solution	SIR-2004 (Direct methods)	
R-Values	wR2 = 0.2993 (R1 = 0.0911 for observed reflections, 0.1748 for all reflections)	
Goodness of fit	S = 1.041	
Max shift/ Error	0.001 * e.s.d	
Largest diff. Peak and hole	0.53, -0.42 $eÅ^{-3}$	

3.4. Crystal data for 4,4'',5,5''-tetradodecyl-2',6'-bis(4-dodecylphenyl)-1,1':5',1''-ternaphthalene (5)

Molecular formula	$C_{114}H_{172}$	
formula weight	1542.51 $gmol^{-1}$	
space group	P -1 (triclinic)	
Absorption	$\mu = 0.42\ mm^{-1}$	
Transmission	$T_{min} = 0.9043$, $T_{max} = 0.9955$	
Crystal size	0.02 x 0.02 x 0.5 mm^3 colorless needles	
Lattice parameters	a = 6.7005(7)Å	α = 76.741(8)°
	b = 18.2040(18)Å	β = 84.464(8)°
	c = 21.190(2)Å	γ = 82.057(8)°
Volume	V = 2486.0(4)$Å^3$	
Z-Value	1	
F(000)	856.0	
Temperature	-143°C	
Density	$d_{x\text{-}ray} = 1.030\ gcm^{-3}$	
Scan type	ω scans	
Scan collection	1°	
Theta collection for data collection	2° ≤ θ ≤ 67.5°	
Limiting indices	-7 ≤ h ≤ 7 -21 ≤ k ≤ 21 -25 ≤ l ≤ 25	
total number of reflections	31313	
Unique number of reflections	8528 ($R_{int} = 0.1057$)	
Observed number of reflections	2635 (|F|/σ(F) > 4.0)	
Structure solution	SIR-2004 (Direct methods)	
R-Values	wR2 = 0.3216 (R1 = 0.0907 for observed reflections, 0.2347 for all reflections)	
Fitgüte	S = 0.859	
Max shift/Error	0.001 * e.s.d	
Largest diff. Peak and hole	0.33, -0.28 $eÅ^{-3}$	
Remarks	Molecule is C_i symmetric	

3.5. Crystal structure of 1-bromo-4,5-didodecylnaphthalene (S3)

The molecule **S3** crystalized in a monoclinic C c unit cell, which contains four molecules. The mean-plane π-π distance between two naphthalene units is 3.61 Å**.** Packing of this molecule shows columnar alignment of the alkyl chains. One alkyl chain is bend, due to the steric hindrance of the other alkyl chain.

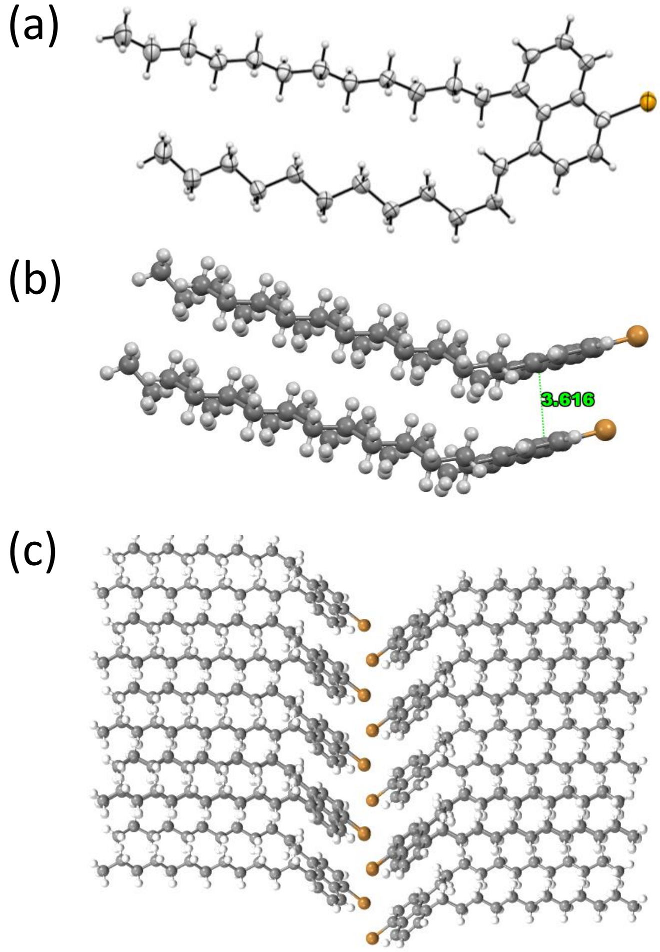

Figure S3. Crystal structure analysis of **S3**. (a) Single molecule arrangement (ORTEP mode); (b) Intermolecular distance between two molecules; (c) Packing viewed along c-axis., (grey: carbon, white: hydrogen, brown; bromo).

3.6. Crystal structure of 2

Single crystal of **2** possesses a triclinic P-1 space group. The trisnaphthalene units are twisted upto 70°, because of high steric hindrance in at the central naphthalene unit. The intermolecular π-π distance averages 6.55 Å but there are C-H...π interactions with 2.85 Å. One alkyl chain is bend, due to the steric hindrance of the other alkyl chain.

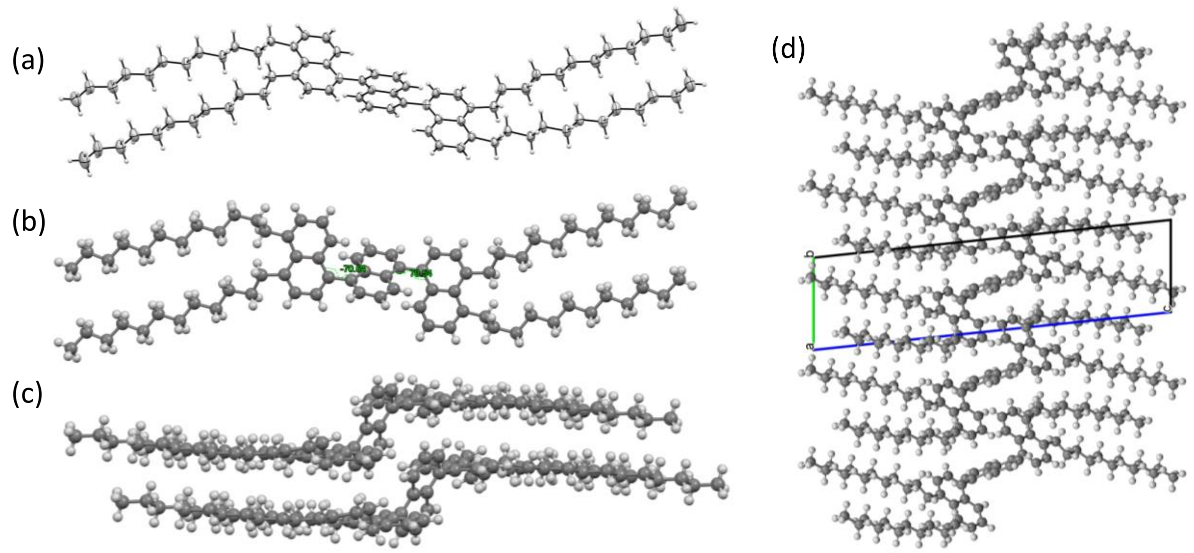

Figure S4. Crystal structure of analysis **2**. (a) Single molecule arrangement (ORTEP mode). (b) π-π interaction between two molecules viewed along b-axis. (c) Packing viewed along b-axis and arrangement of second layer. (d) Packing viewed along c-axis, (grey: carbon, white: hydrogen, red: oxygen, green: fluorine, yellow: sulfur).

3.7. Crystal structure of 4

Single crystal of **4** possesses a triclinic P-1 space group. The trisnaphthalene units are twisted upto 90°, because of high steric hindrance in at the central naphthalene unit. The intermolecular π-π distance averages 3.65 Å. Here the same situation of the arrangement of the alkyl chains applied. One alkyl chain is bend, due to the steric hindrance of the other alkyl chain. The bond lengths from carbon to oxygen account 1.43 Å, from oxygen to sulfur 1.56 Å, from sulfur to carbon attached to three fluorine atoms 1.84 Å and from carbon to the attached fluorine atoms 1.30 Å. The Sulfur atom with the three oxygen atoms are arranged trigonal planar. The three fluorine atoms attached to the carbon atom are arranged in a trigonal pyramidal geometry.

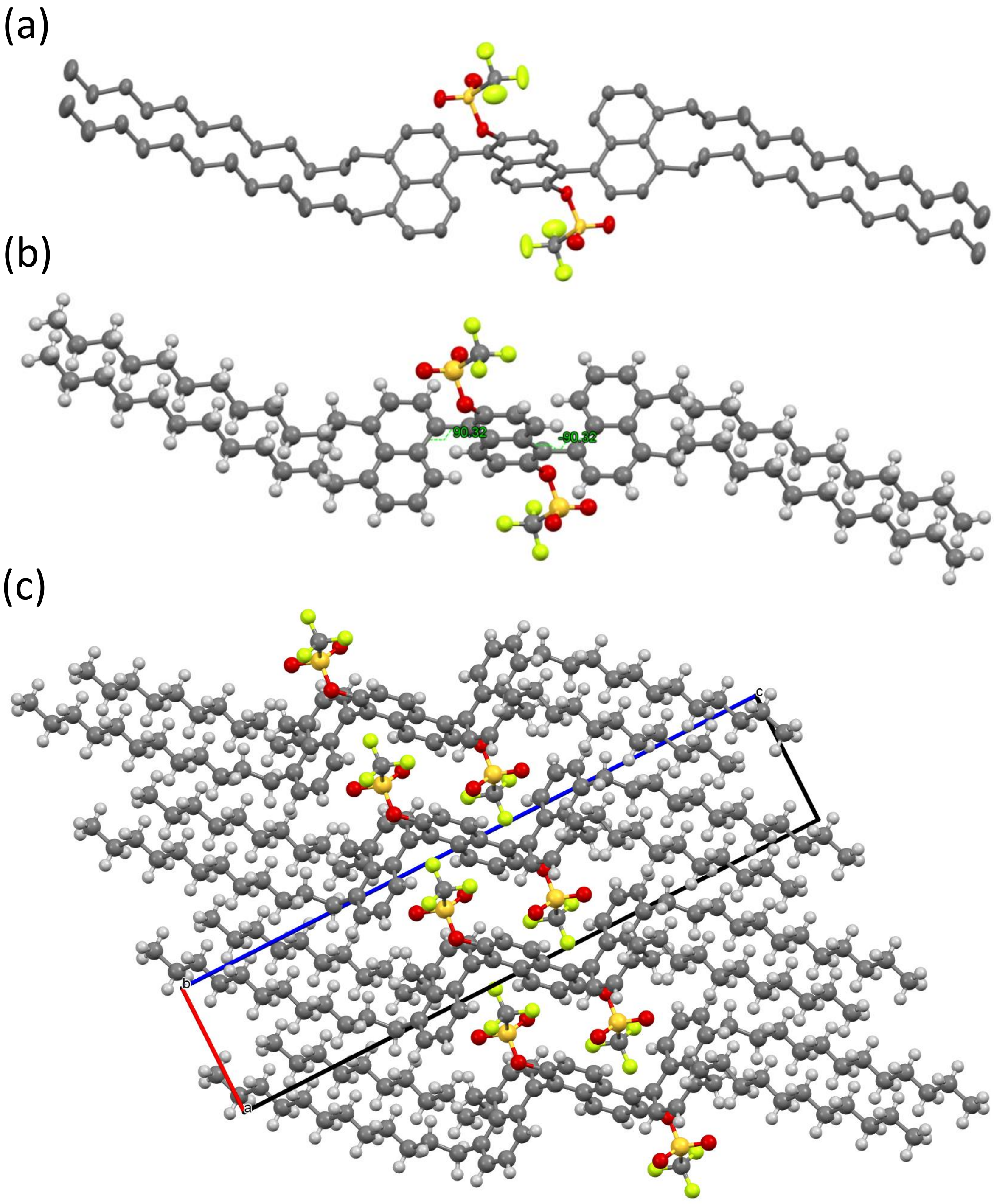

Figure S5. Crystal structure of analysis **4**. (a) Single molecule arrangement (ORTEP mode). (b) π-π interaction between two molecules viewed along b-axis. (c) Packing viewed along b-axis and arrangement of second layer. (d) Packing viewed along c-axis, (grey: carbon, white: hydrogen, red: oxygen, green: fluorine, yellow: sulfur).

3.8. Crystal structure of 5

Single crystal of **5** possesses a triclinic P-1 space group. The trisnaphthalene units are twisted upto 102°, because of high steric crowding on the central naphthalene unit. There are C-H...π interactions with 2.85 Å. Here the same situation of the arrangement of the alkyl chains applied. One alkyl chain is bend, due to the steric hindrance of the other alkyl chain.

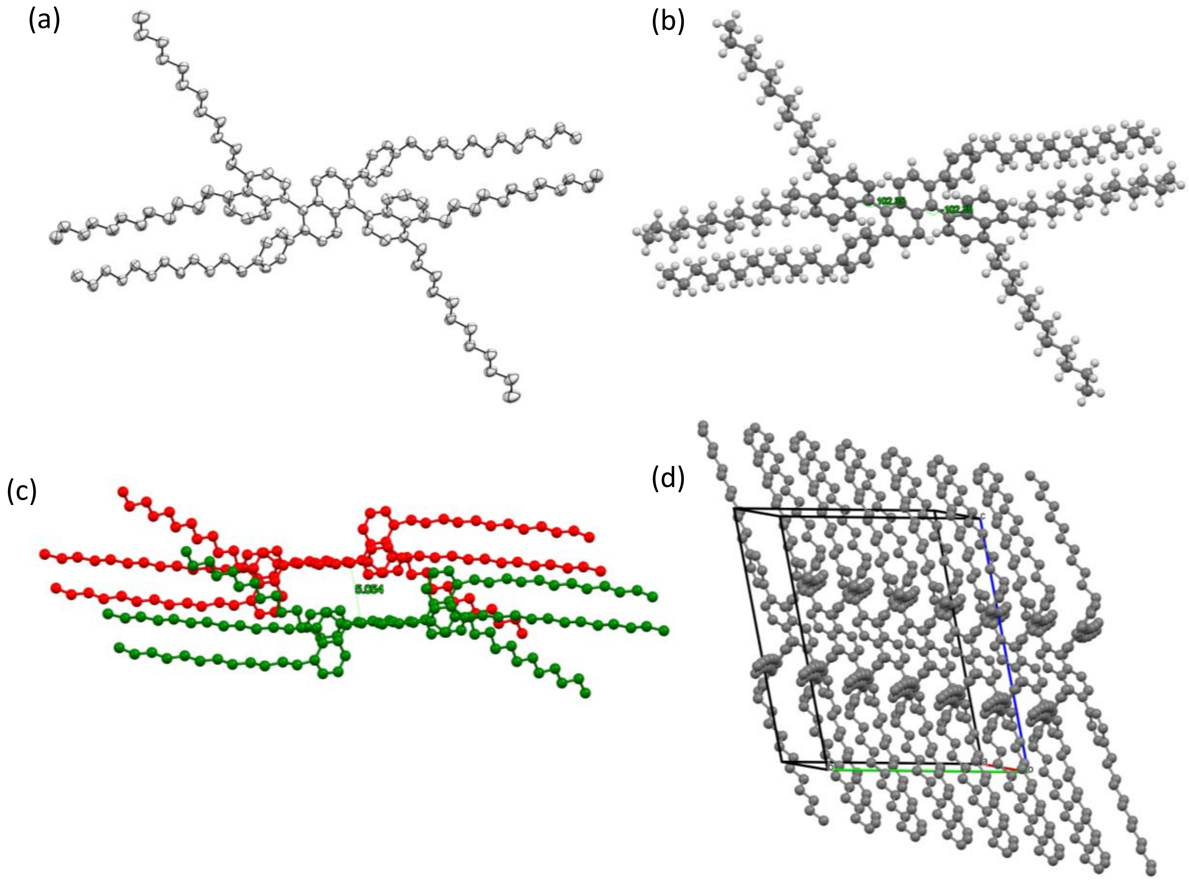

Figure S6. Crystal structure of analysis **5**. (a) Single molecule arrangement (ORTEP mode). (b) π-π interaction between two molecules viewed along b-axis. (c) Packing viewed along b-axis and arrangement of second layer. (d) Packing viewed along c-axis, (grey: carbon, white: hydrogen).

4. Differential scanning calorimetry (DSC):

Differential scanning calorimetry (DSC) were measured on a Mettler DSC 30 with heating and cooling rates of 10 K/min.

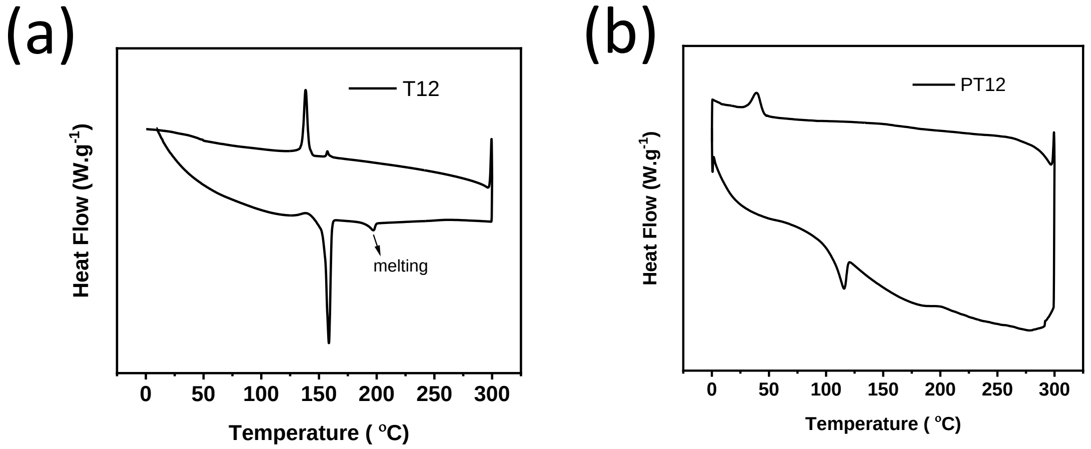

Figure S7. DSC curves (**a**) of *peri*-alkylated terrylene **T12** and (**b**) phenylene-fused terrylene **PT12**.

5. Photophysics and transient absorption and photoluminescence spectroscopy:

Toluene solutions of both molecules were prepared in an oxygen free glovebox environment. A 0.22µm PTFE filter was used to ensure the removal of any precipitates. The peri-alkylated terrylene (**T12**) was made up to a concentration of 7.5µM and phenylene-fused terrylene (**PT12**) to 10µM for the steady-state and transient optical characterisation experiments. Time-correlated single photon counting (TCSPC) was performed on Edinburgh Instruments FLS1000 photoluminescence spectrometer using pulsed excitation from an Edinburgh Instruments Agile Picosecond Pulsed Light Source. This data was interpreted by tail fitting the time resolved photoluminescence profile to the following first order exponential:

$$Counts = Ae^{\frac{-t}{\tau}}$$

Photoluminescence quantum yield (Φ_F) measurements were performed on Hamamatsu Quantaurus-QY PL quantum yield spectrometer.

Transient absorbance (TA) experiments were conducted using a Light Conversion HARPIA Ultrafast Spectroscopy system with sample excitation from a femtosecond Light Conversion Pharos 1030 nm pump laser and a Light Conversion ORPHEUS Colinear Optical Parametric Amplifier. Sample excitation fluence of 4.3 mJ cm^{-2} and 5.2 mJ cm^{-2} was used for T12 and

PT12, respectively, to collect data shown in manuscript. The same sample kinetics were observed for TA experiments using 1/10 and 1/100 excitation fluence, meaning the experiments reflect monomolecular photophysics.

TA data were fitted to time constants according to the following equation:

$$M(t;\boldsymbol{A},\boldsymbol{\tau}) = \frac{\sqrt{4ln(2)}}{\sqrt{\pi}FWHM} e^{-\frac{4\cdot ln(2)\cdot(t-D_0)^2}{FWHM^2}} \times \left\{ \mathrm{H}(\mathrm{t}-D_0)\cdot \sum_{i=1}^{ncomp} A_i e^{-\frac{t-D_0}{\tau_i}} \right\}$$

Table S**2**. All visible TA features were fitted to the same two time constants for each molecule.

Property measured	***T12***	***PT12***
τ_1	4.2 ns	3.6 ns
τ_2	540 ps	540 ps

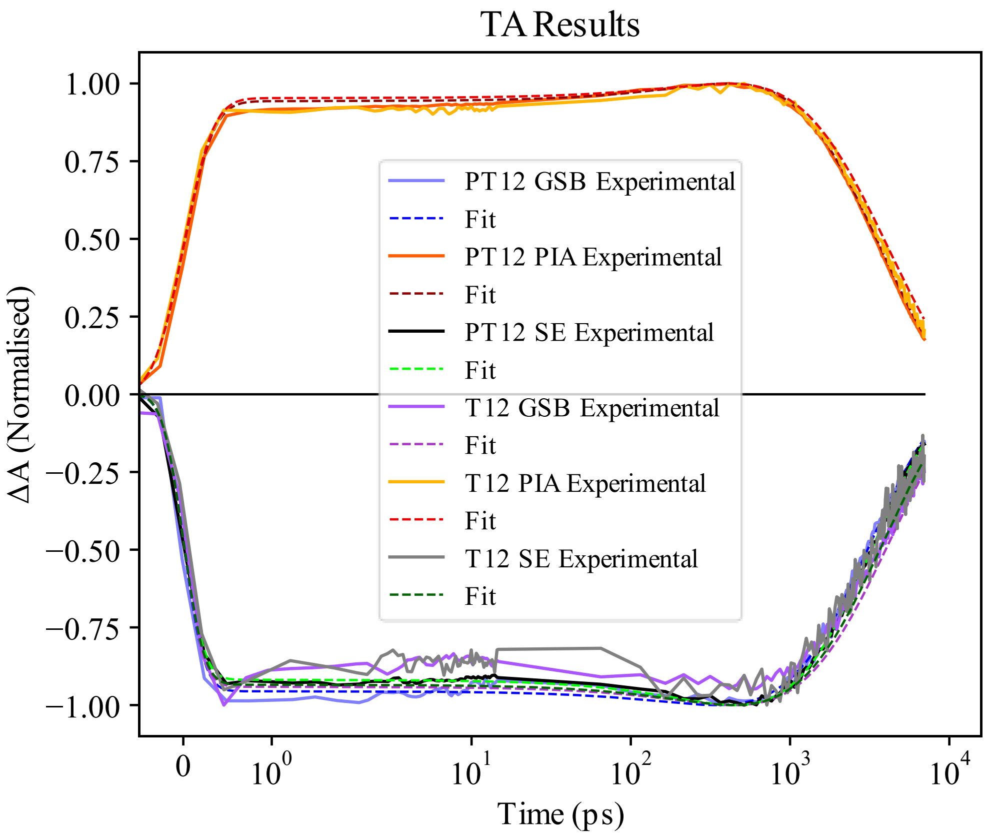

Figure S8. Transient absorbance (TA) spectroscopy excited state dynamics for both **T12** and **PT12** upon excitation at 584 nm and 537 nm, respectively.

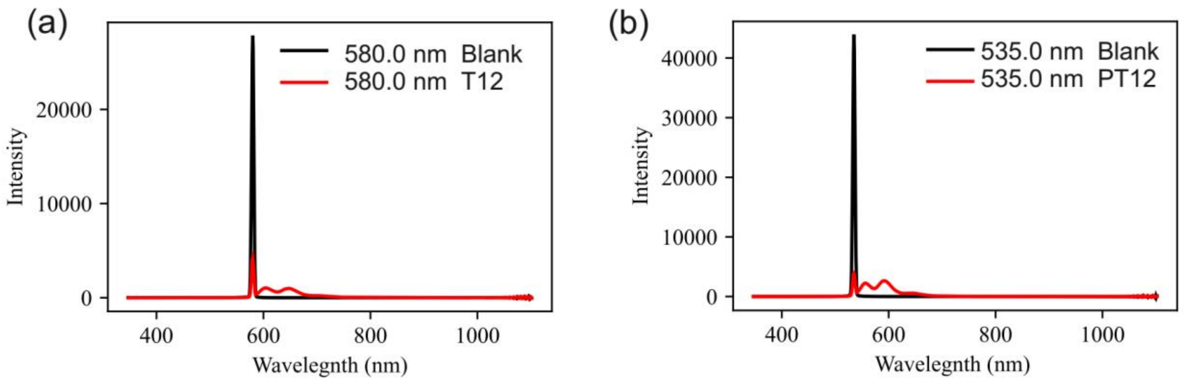

Figure S9. Photoluminescence quantum yield studies of **T12** and **PT12** were conducted with 580 nm and 535 nm excitation at the low energy absorption edge of the materials.

6. Density functional theory (DFT) calculations:

DFT were carried out at the B3LYP/6-31G (d) level using Gaussian 09[44] and molecular structures were generated using GaussView 5.0.9.[45] The side chains were set to CH_3 (methyl) to simplify the calculation.

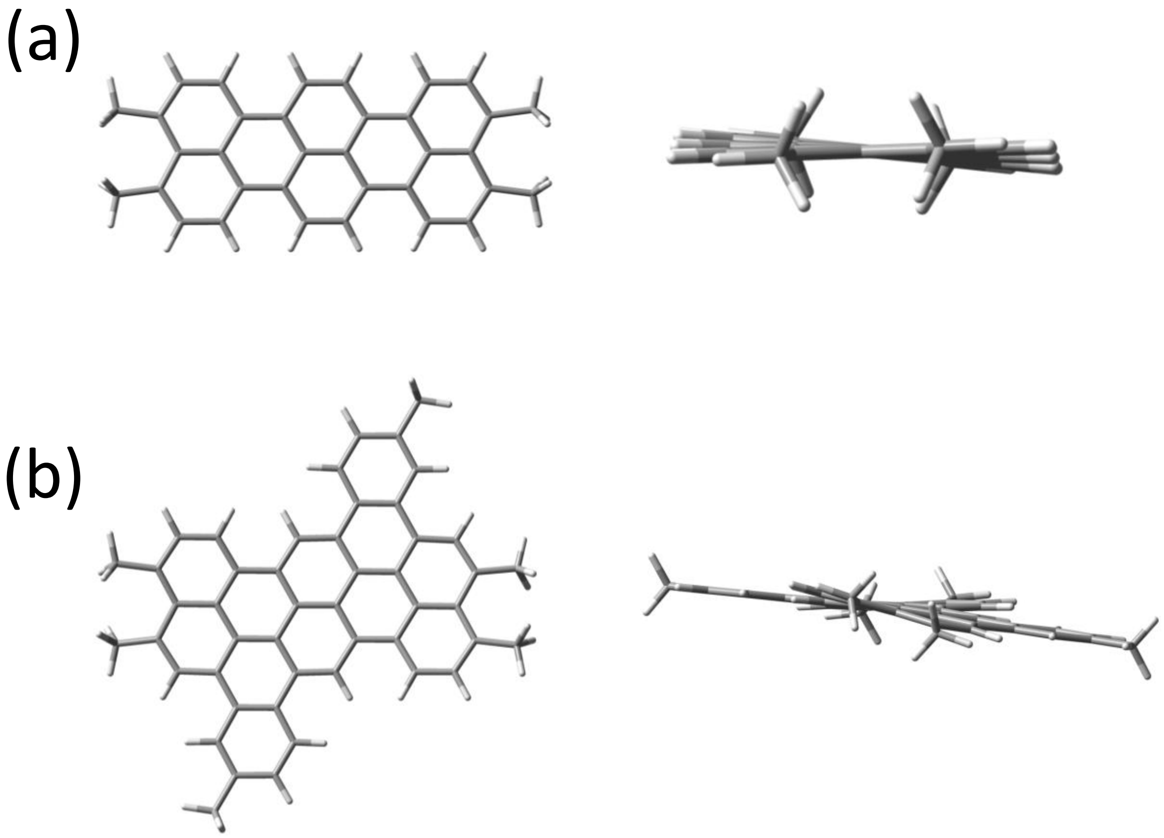

Figure S10. Geometry and energy optimized chemical structures (**a**) of *peri*-alkylated terrylene **T12** and (**b**) phenylene-fused terrylene **PT12**.

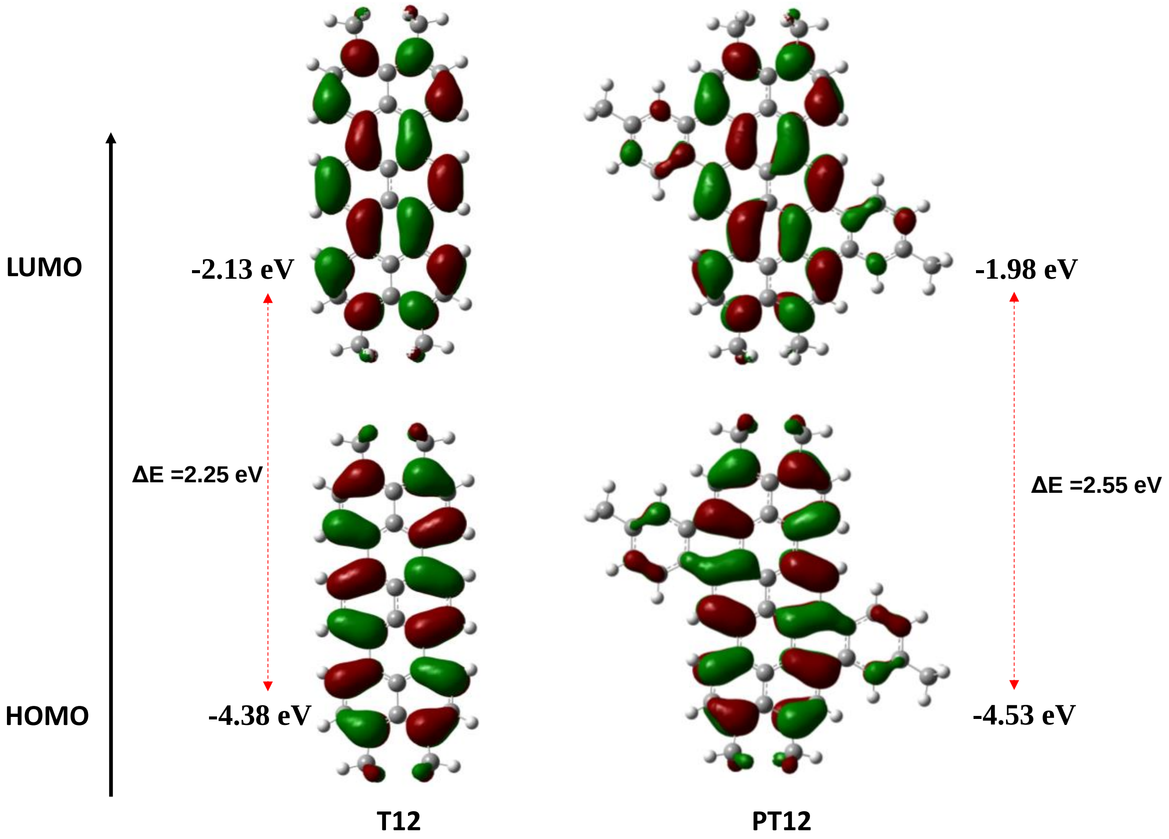

Figure S11. calculated (B3LYP/6-31G*) HOMO and LUMO profiles of the terrylene molecules, **T12** and **PT12**.

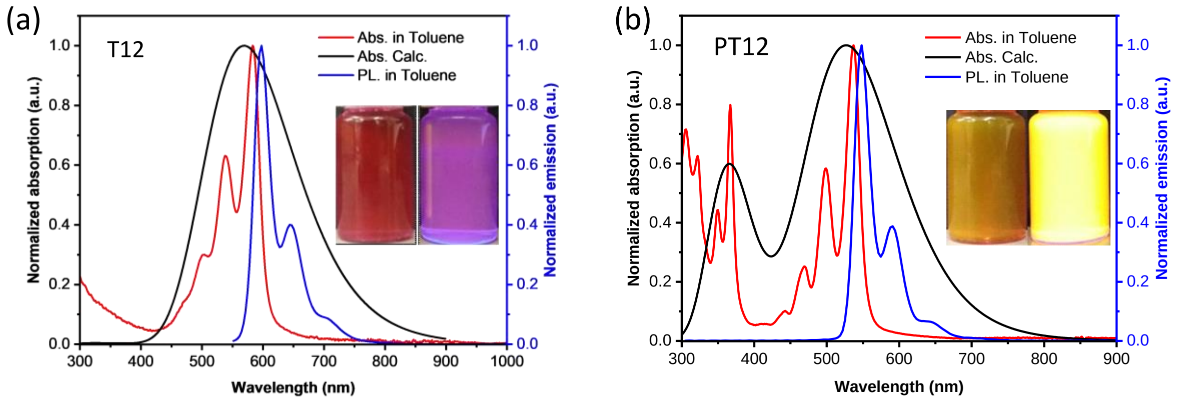

Figure S12. Absorption and photoluminescence spectra of (a) **T12** and (b) **PT12**. Insets in each panel are optical images of dilute solutions of molecules under white light (left) and UV light exposure.

7. ^{1}H-NMR, ^{13}C-NMR spectra of synthesized compounds

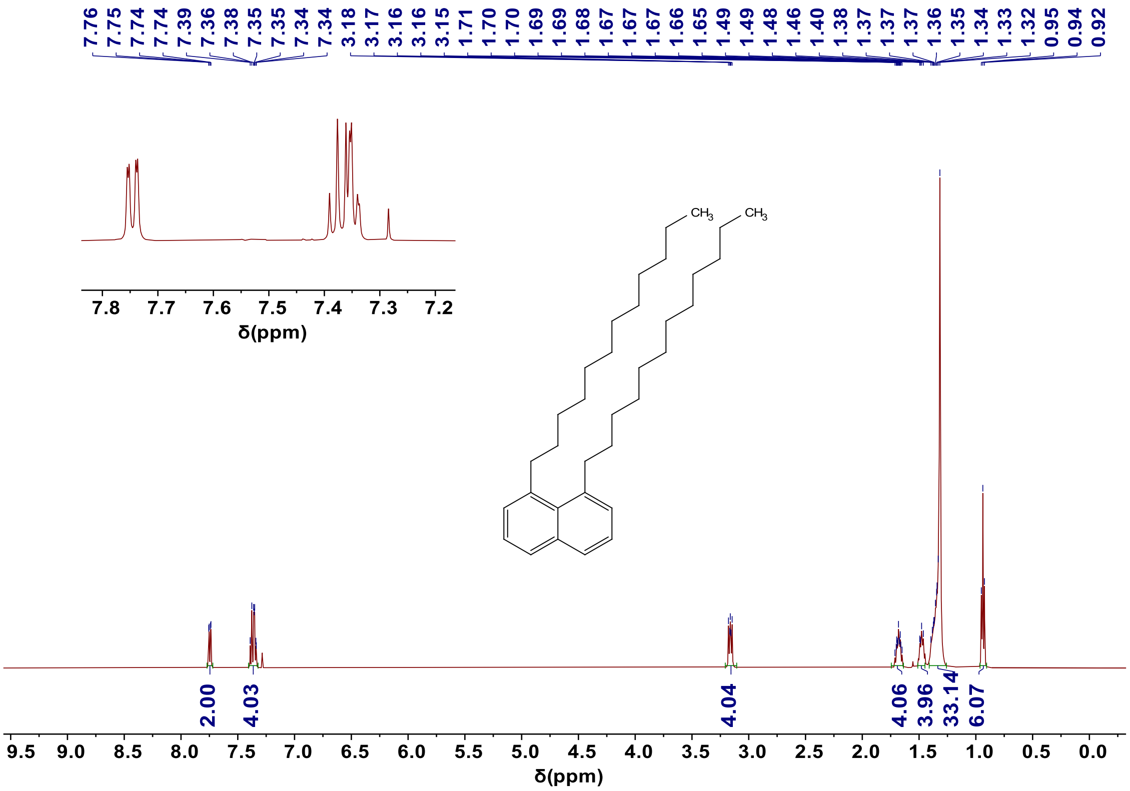

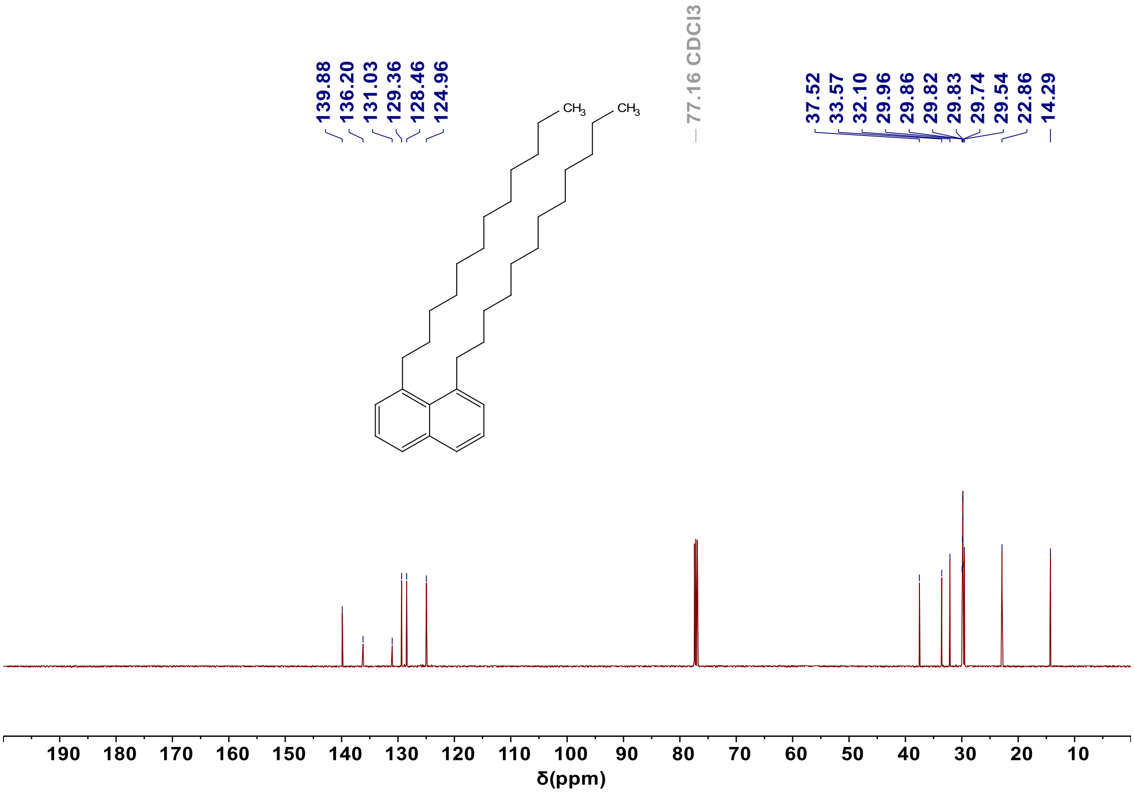

Figure S13. ^{1}H NMR and ^{13}C NMR spectrum of compound **S2.**

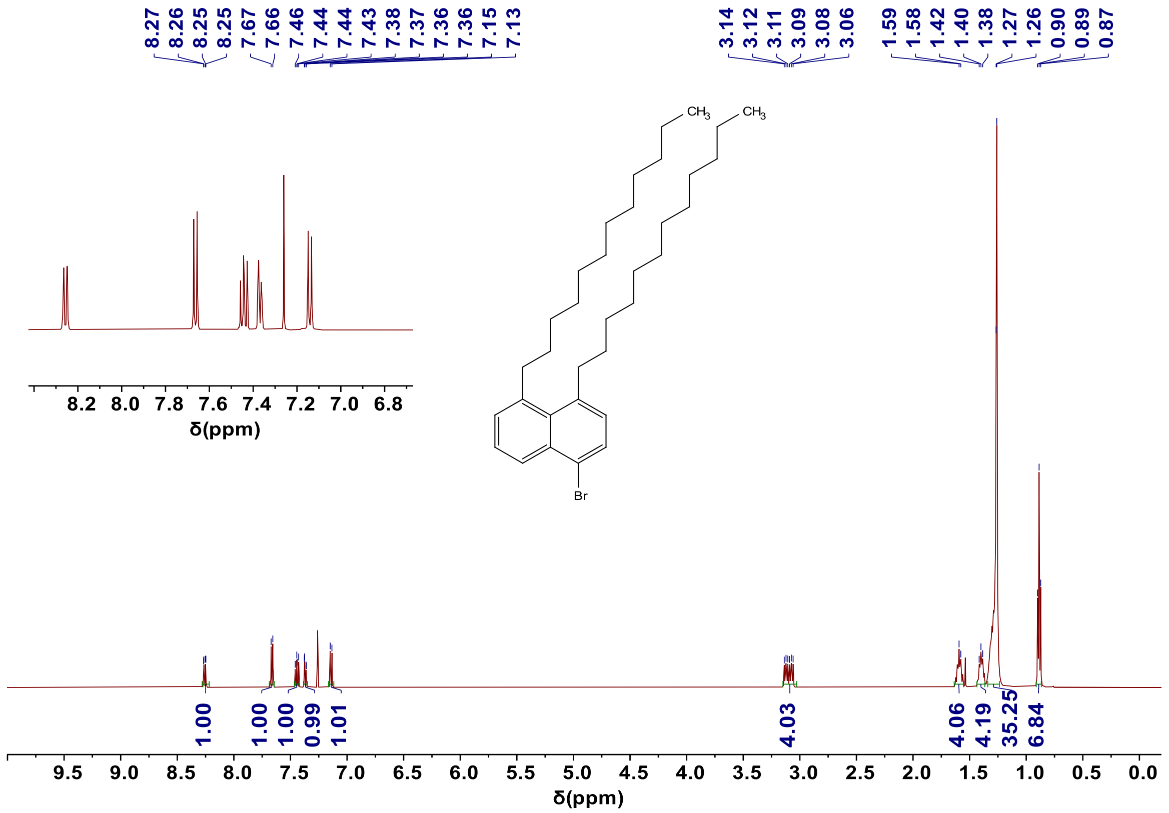

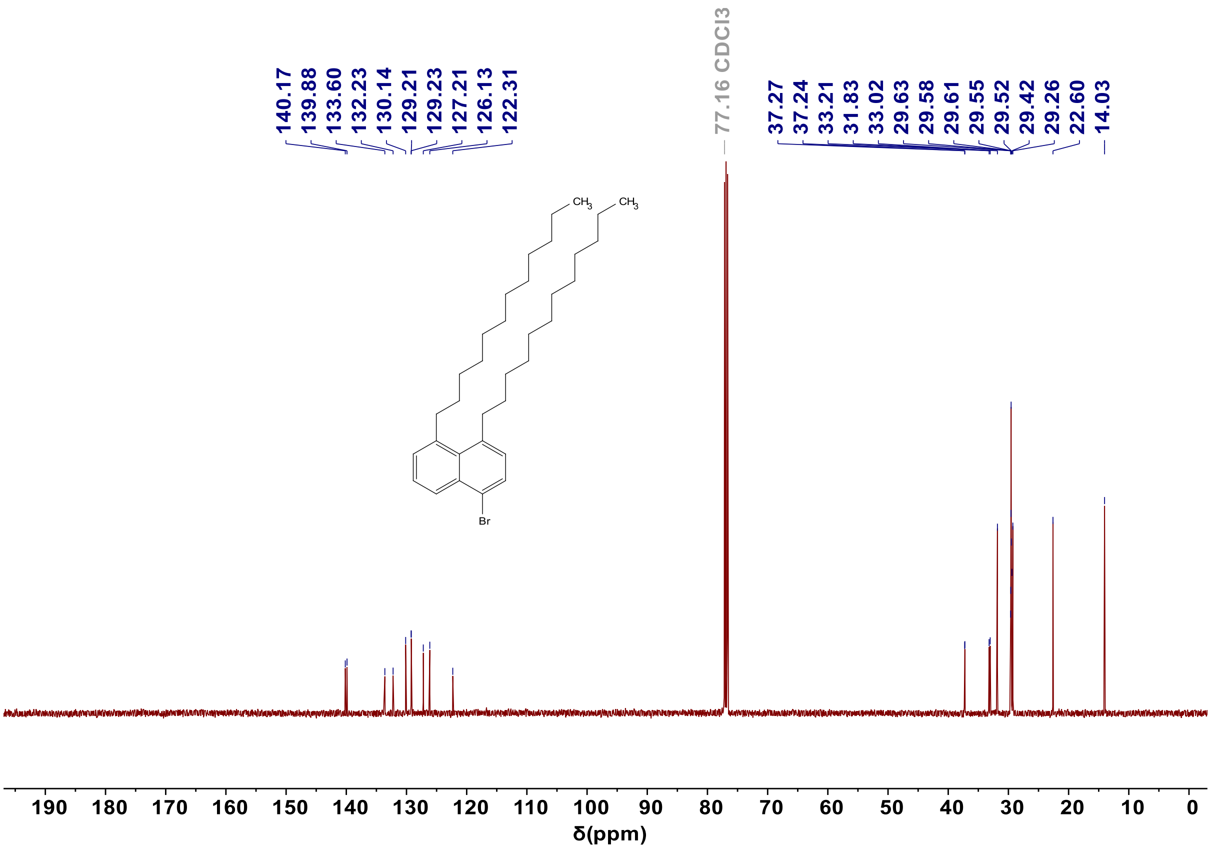

Figure S14. ^{1}H NMR and ^{13}C NMR spectrum of compound **S3.**

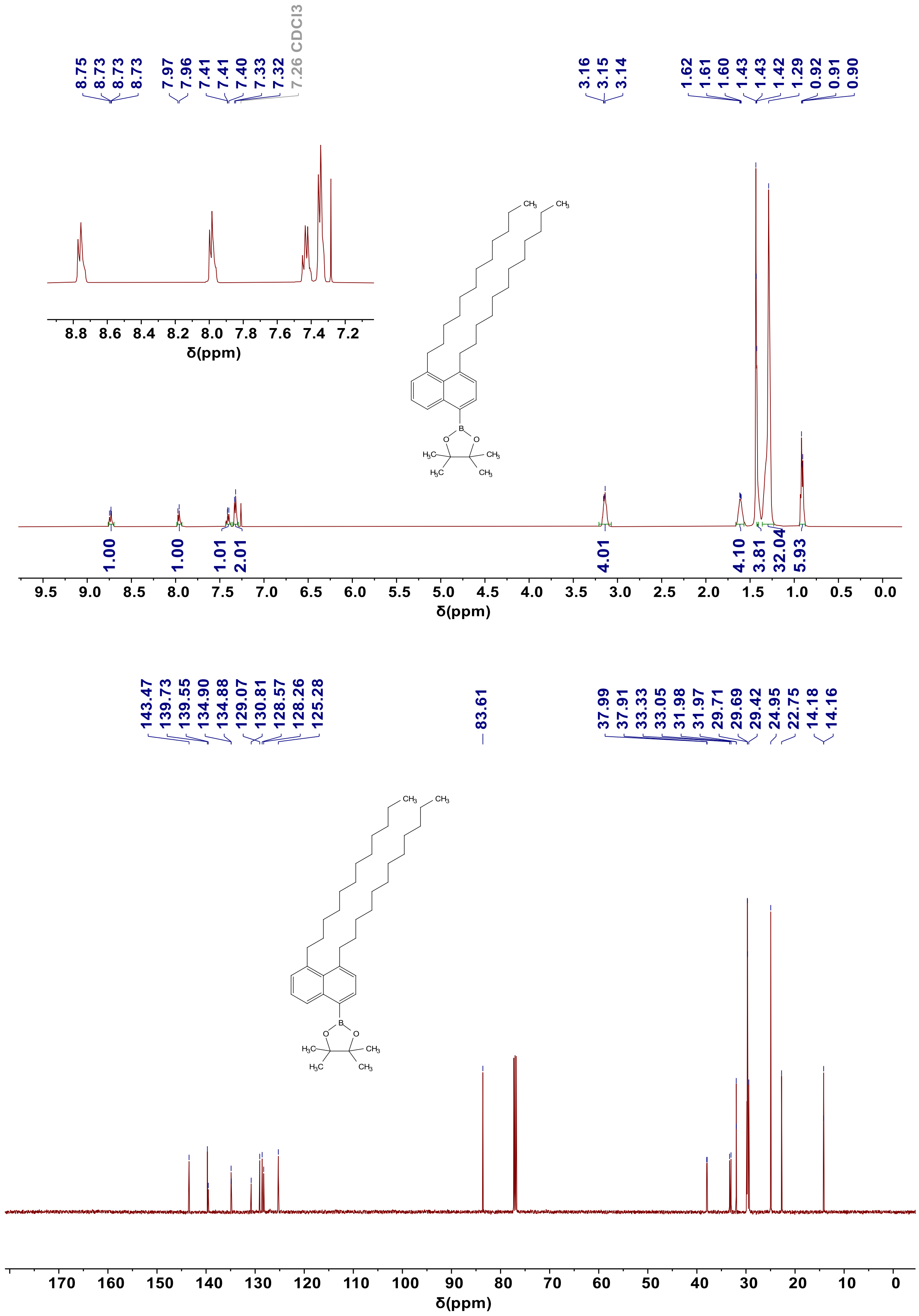

Figure S15. ^{1}H NMR and ^{13}C NMR spectrum of compound **1.**

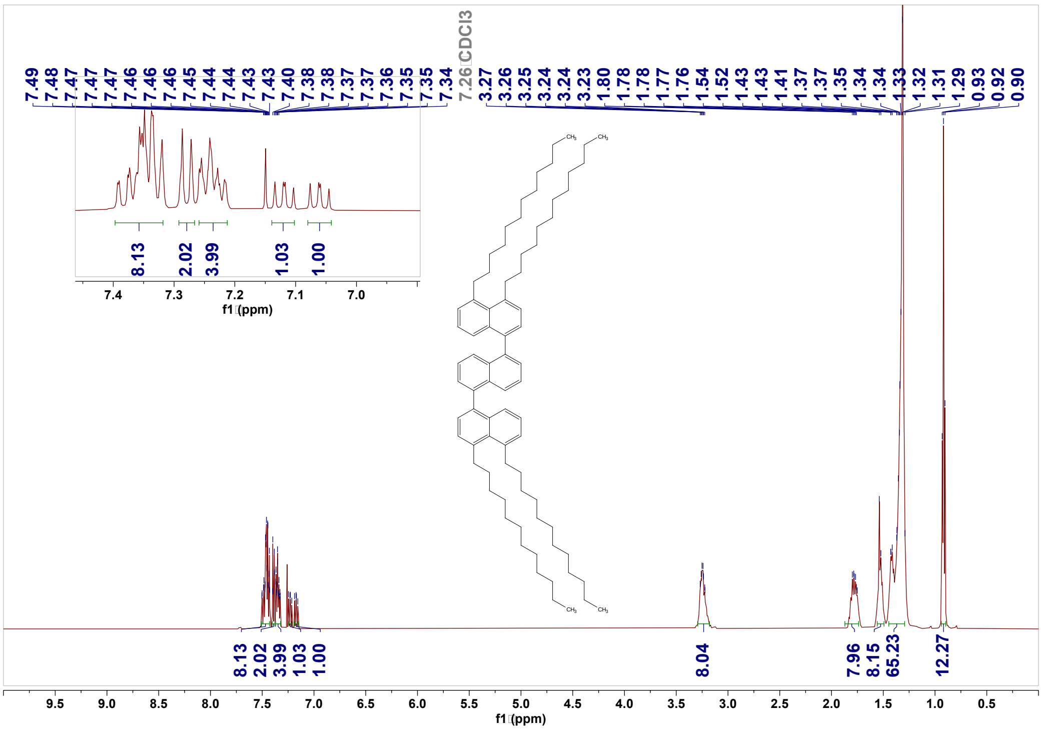

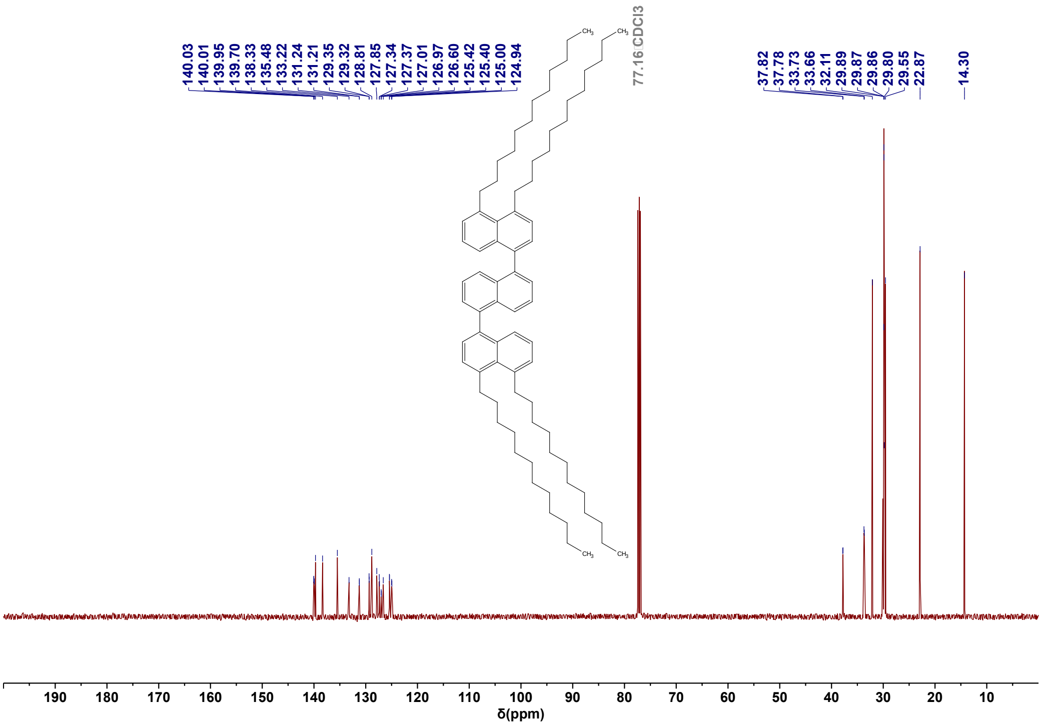

Figure S16. ^{1}H NMR and ^{13}C NMR spectrum of compound **2.**

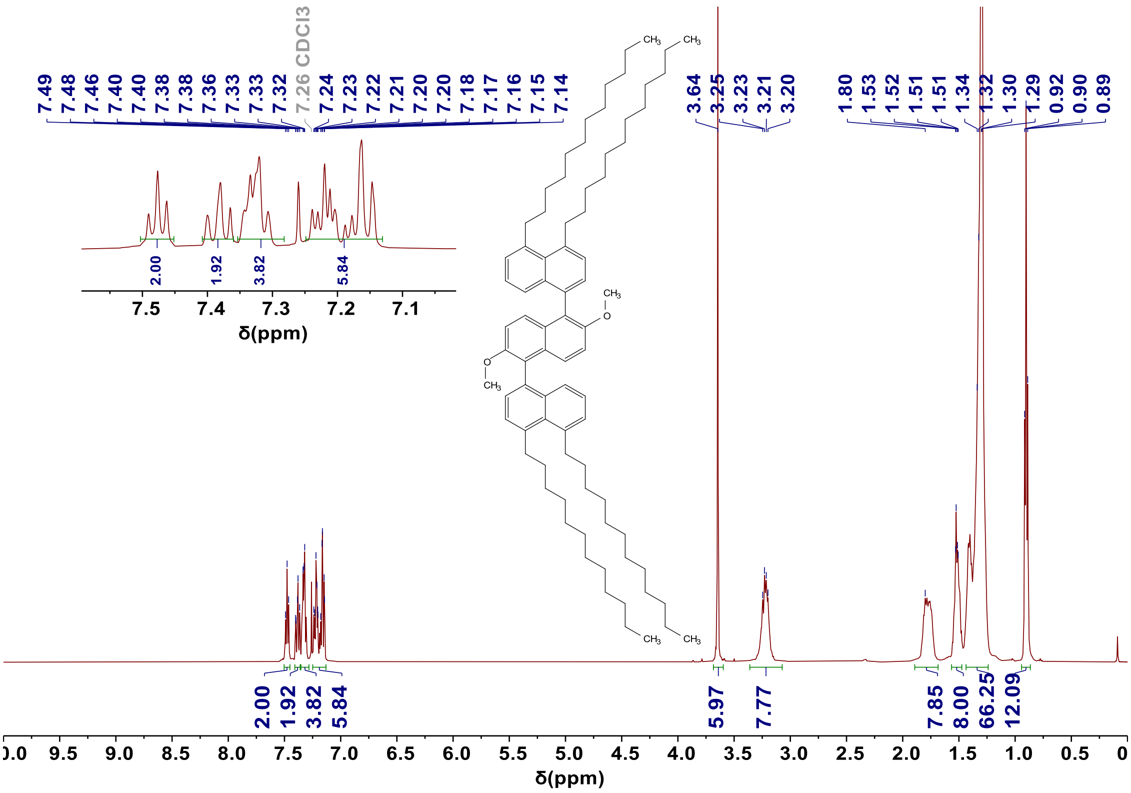

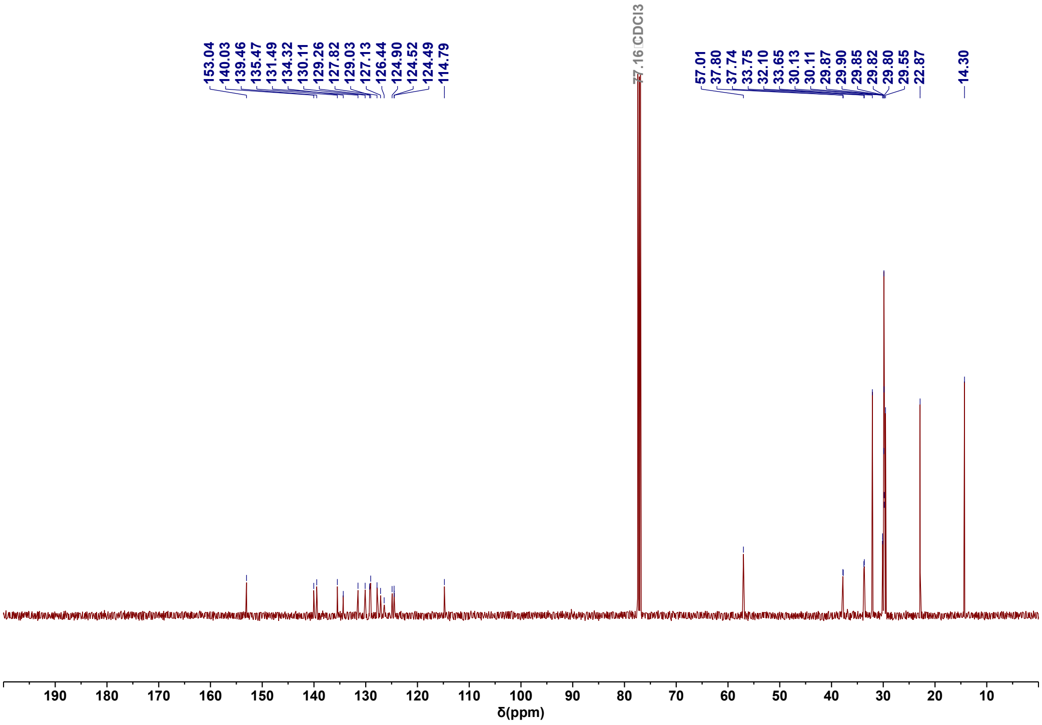

Figure S17. ^{1}H NMR and ^{13}C NMR spectrum of compound **3.**

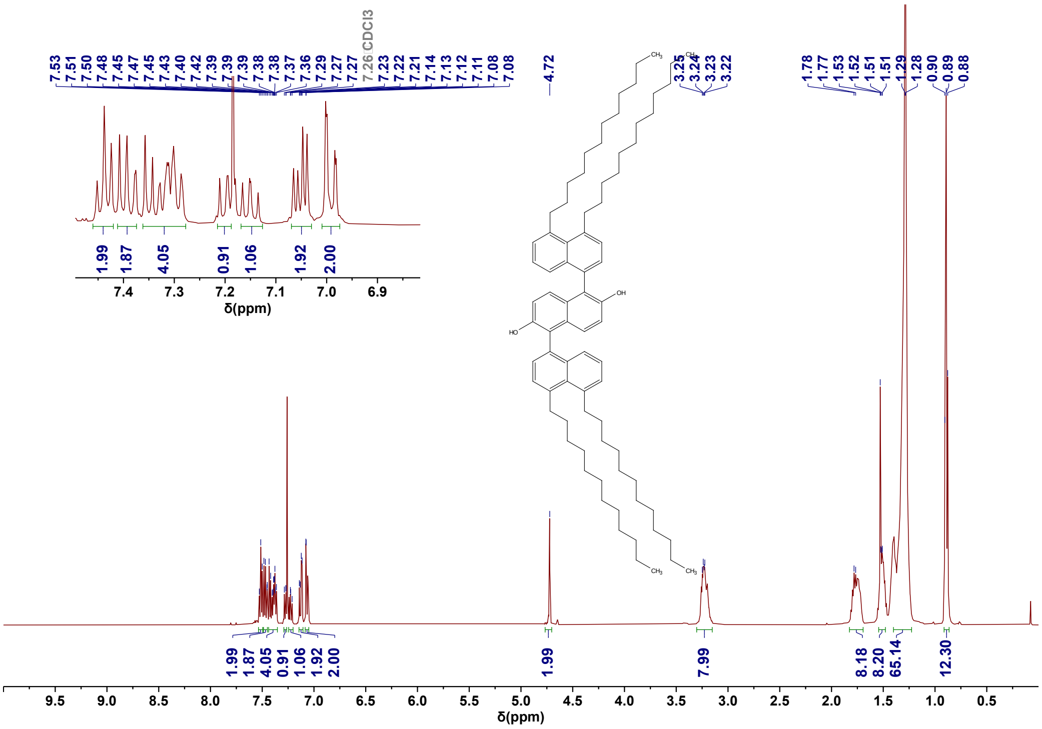

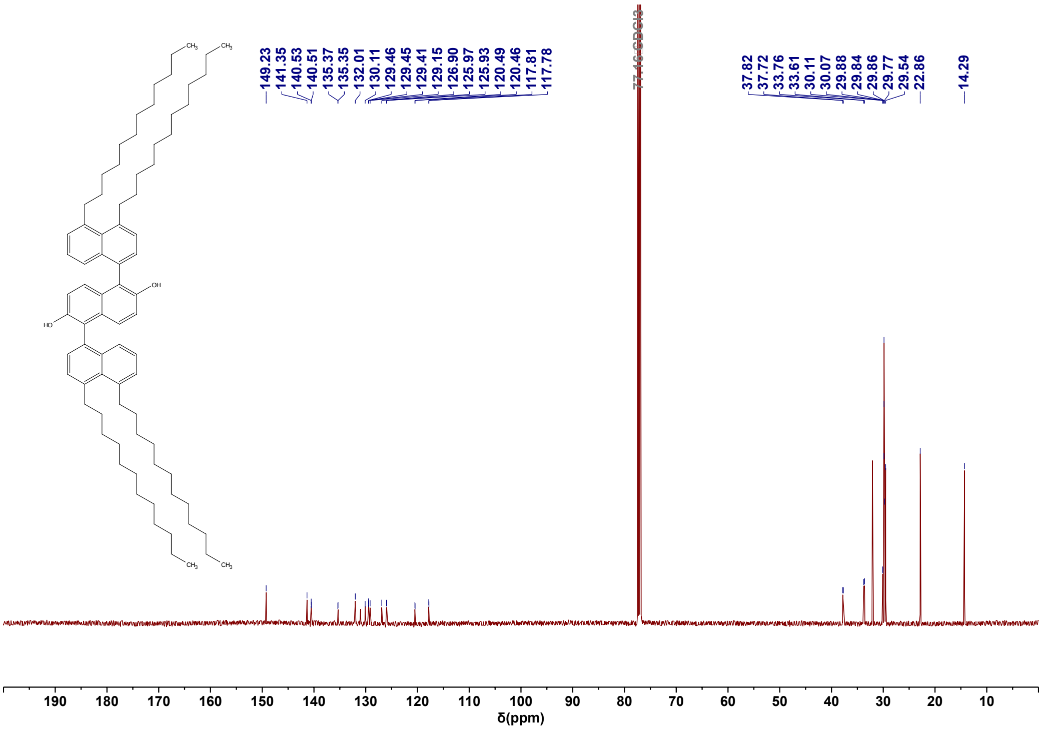

Figure S18. ^{1}H NMR and ^{13}C NMR spectrum of compound **4'.**

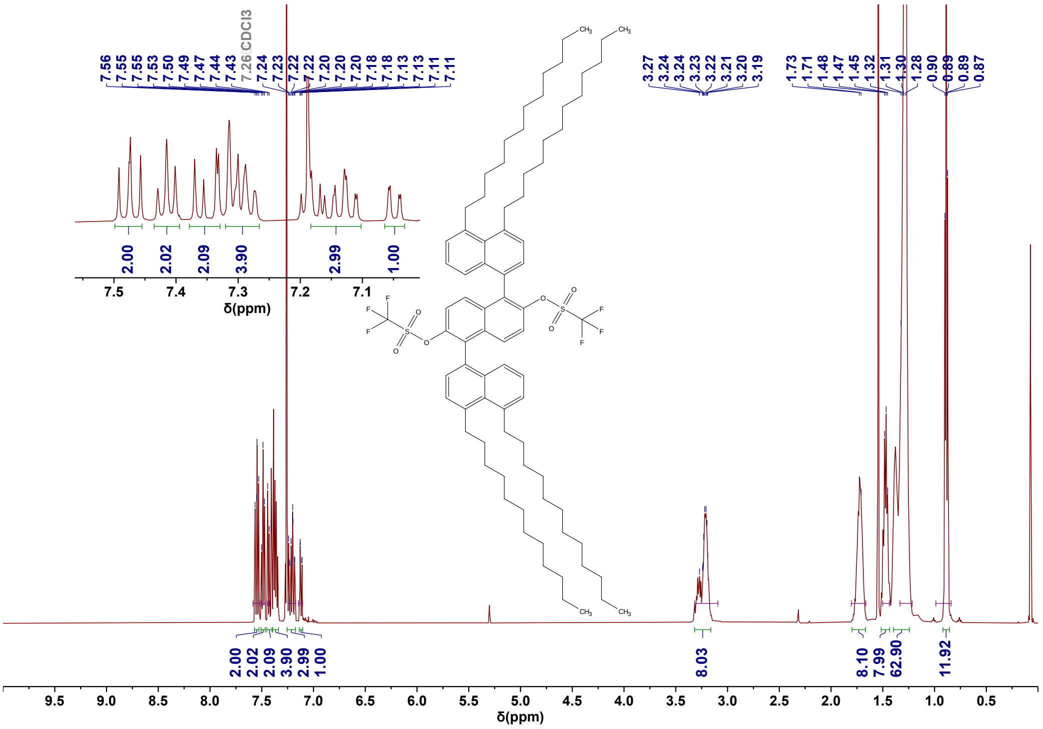

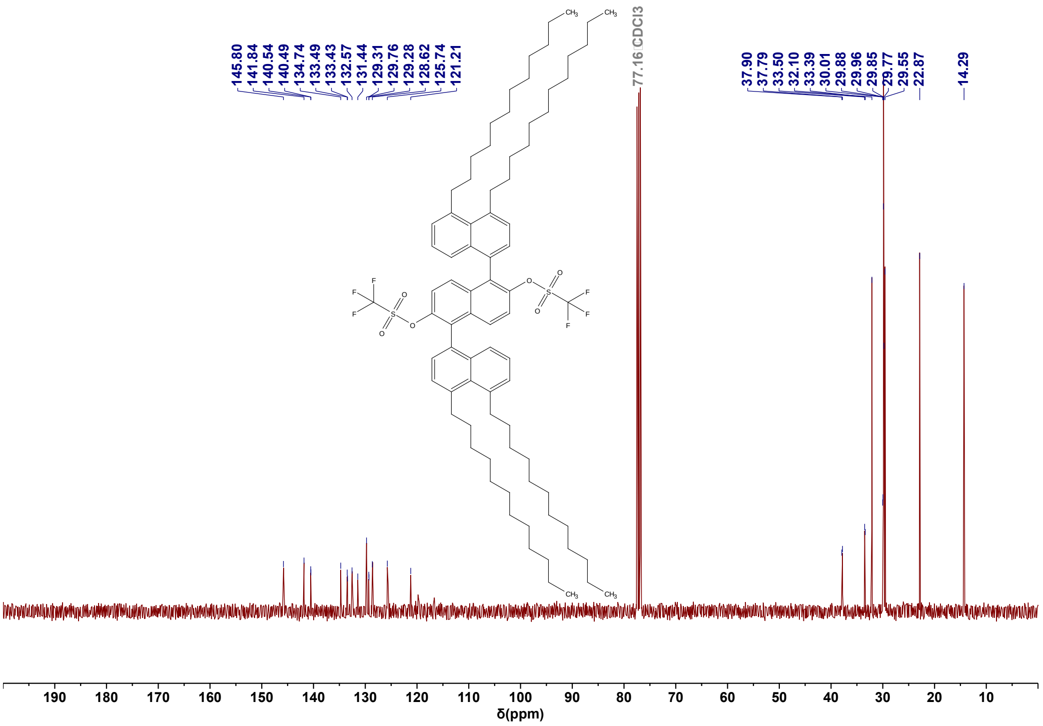

Figure S19. ^{1}H NMR and ^{13}C NMR spectrum of compound **4.**

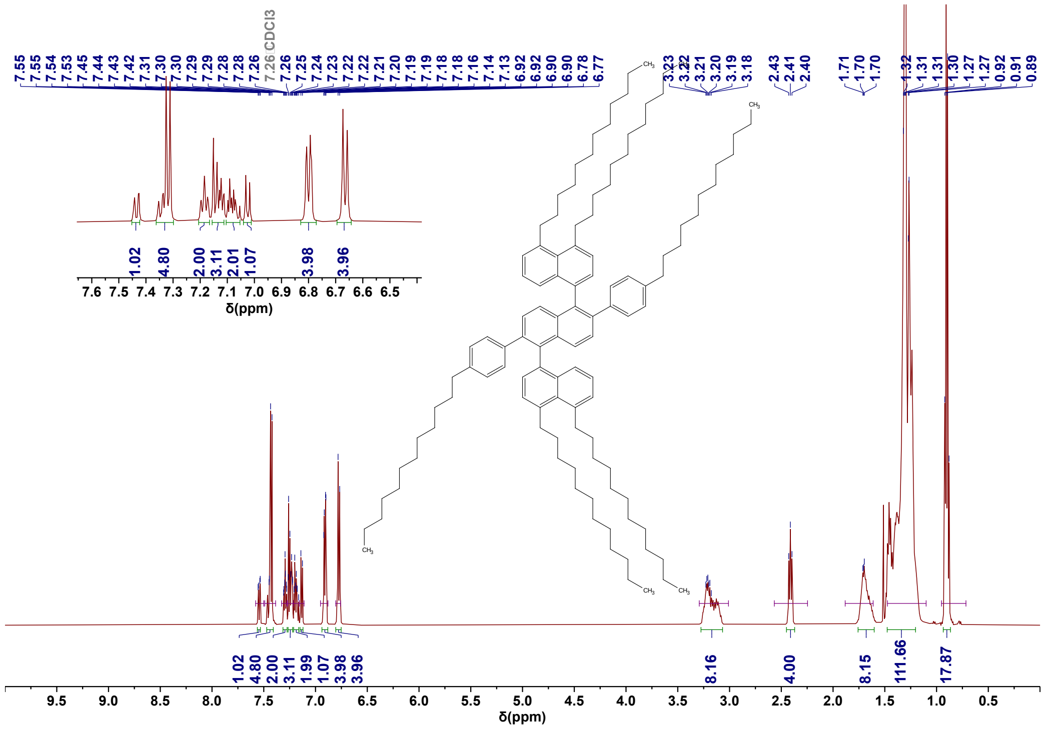

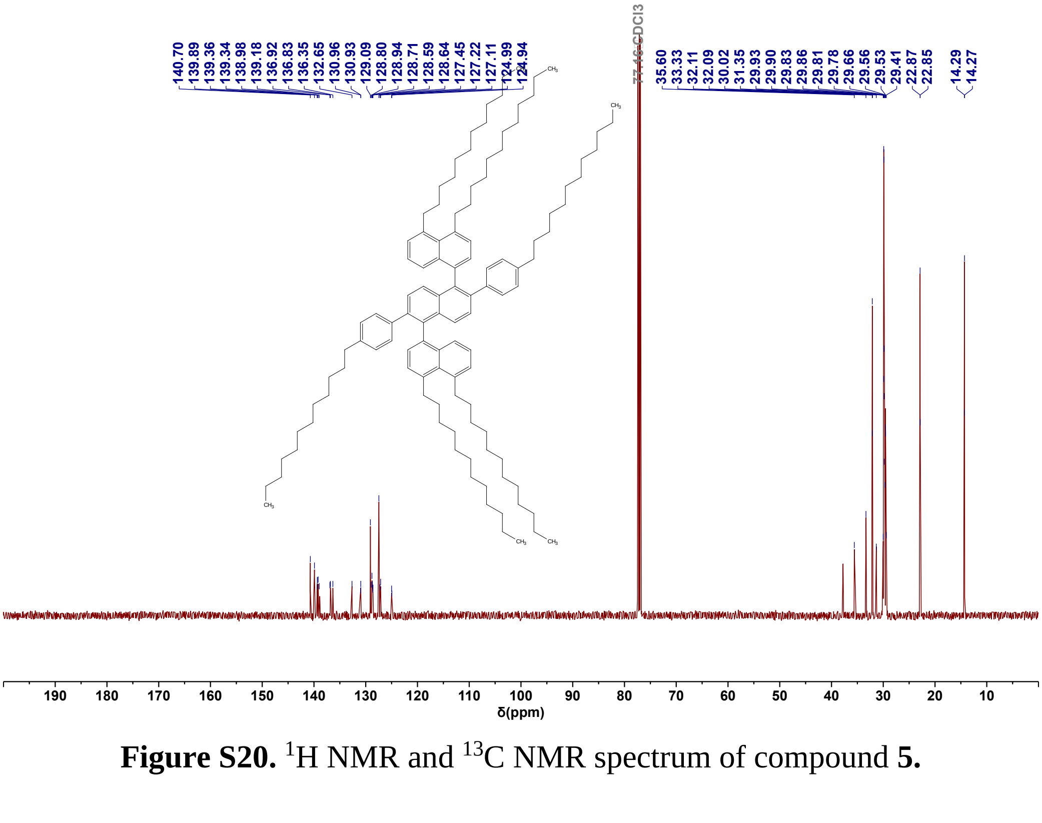

Figure S20. ^{1}H NMR and ^{13}C NMR spectrum of compound **5.**

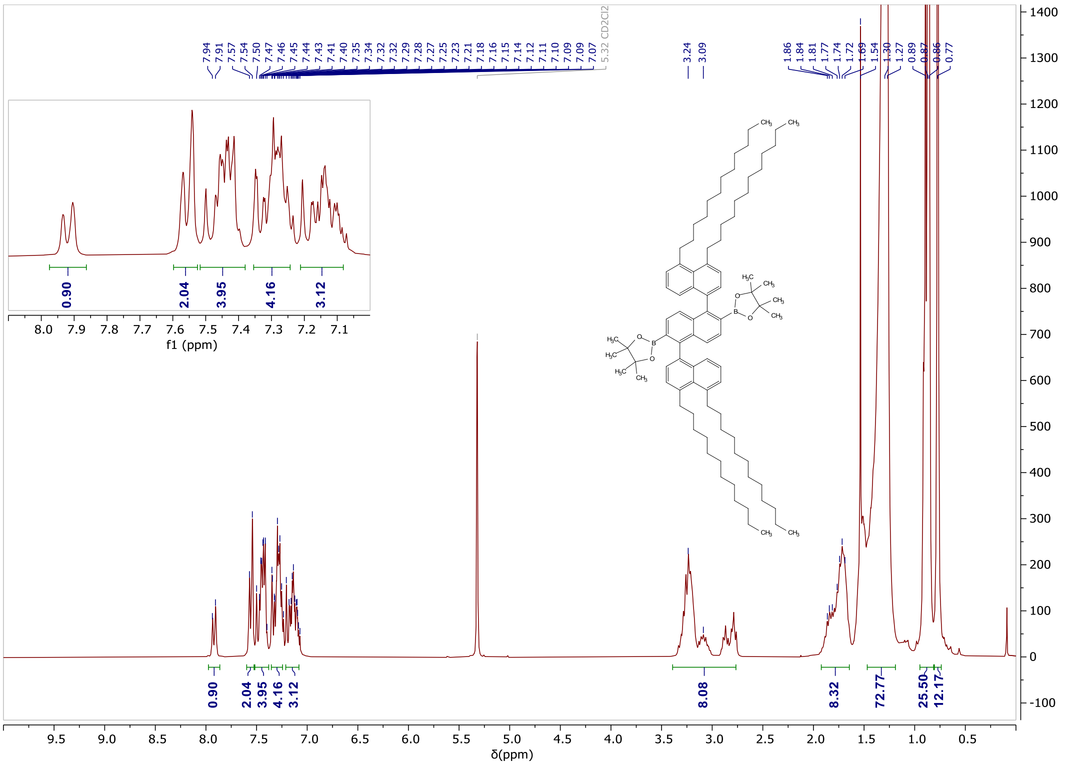

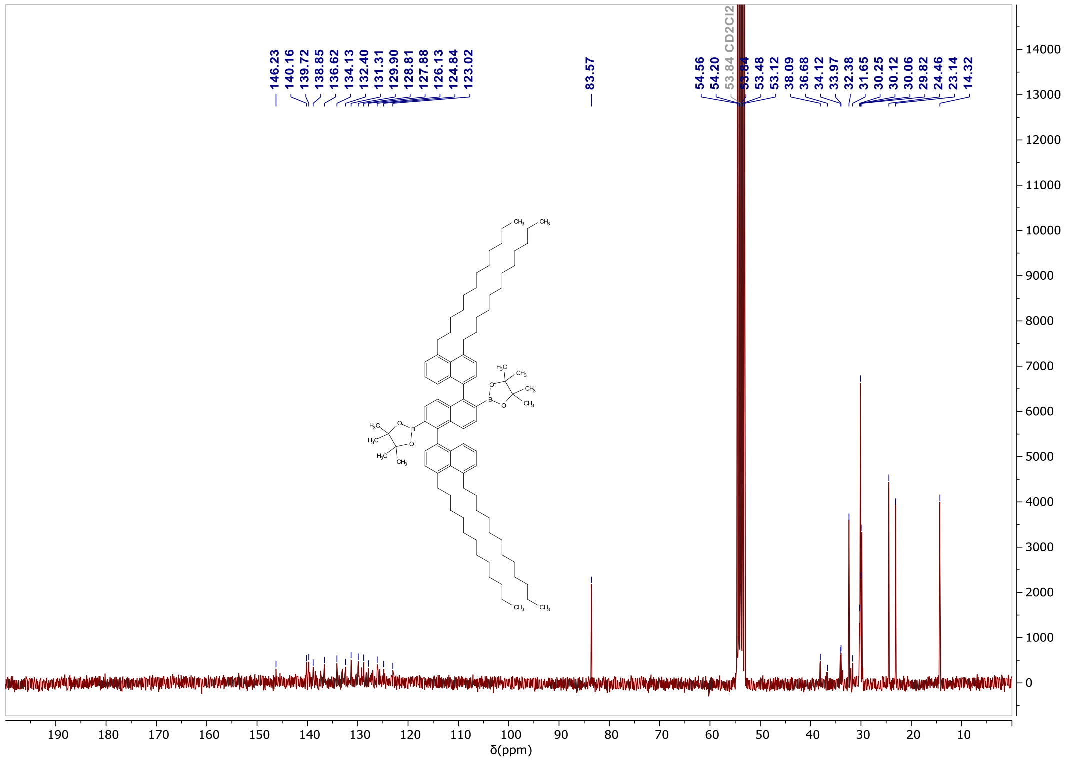

Figure S21. ^{1}H NMR and ^{13}C NMR spectrum of compound **6.**

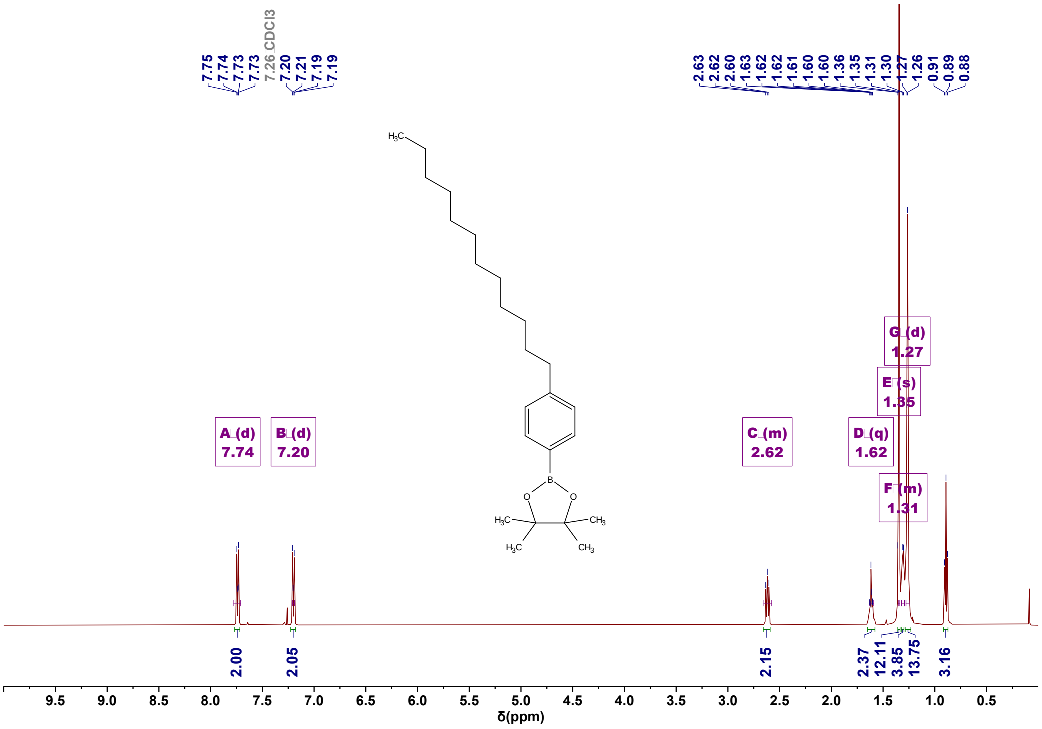

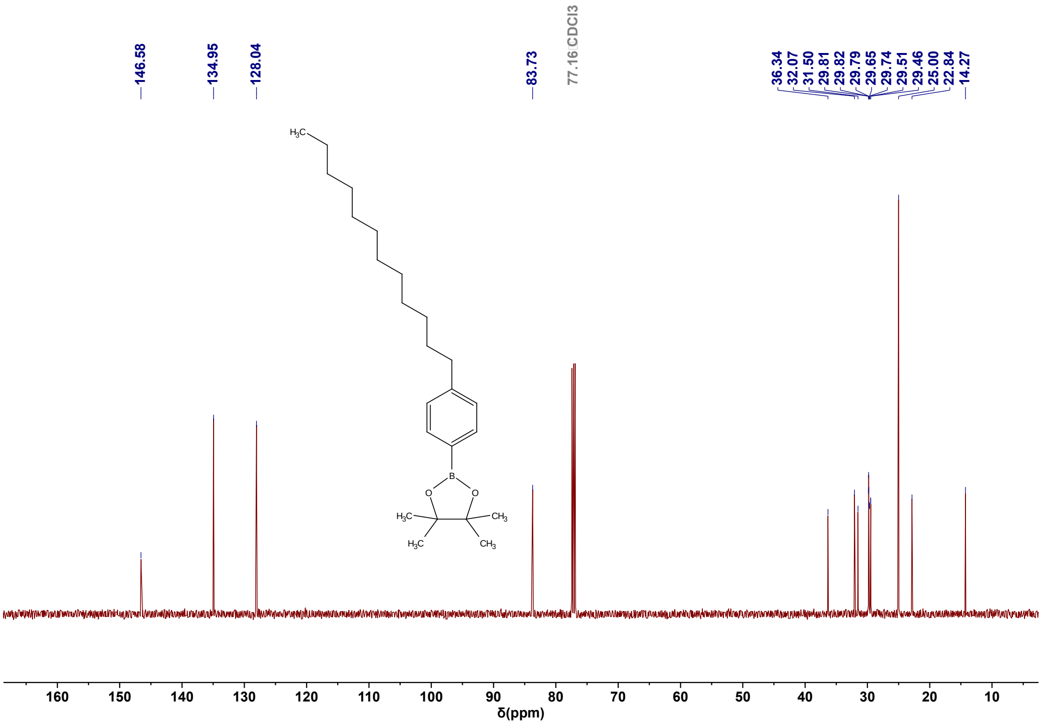

Figure S22. ^{1}H NMR and ^{13}C NMR spectrum of **2-(4-Dodecylphenyl)-4,4,5,5-tetramethyl-1,3,2-dioxaborolane.**

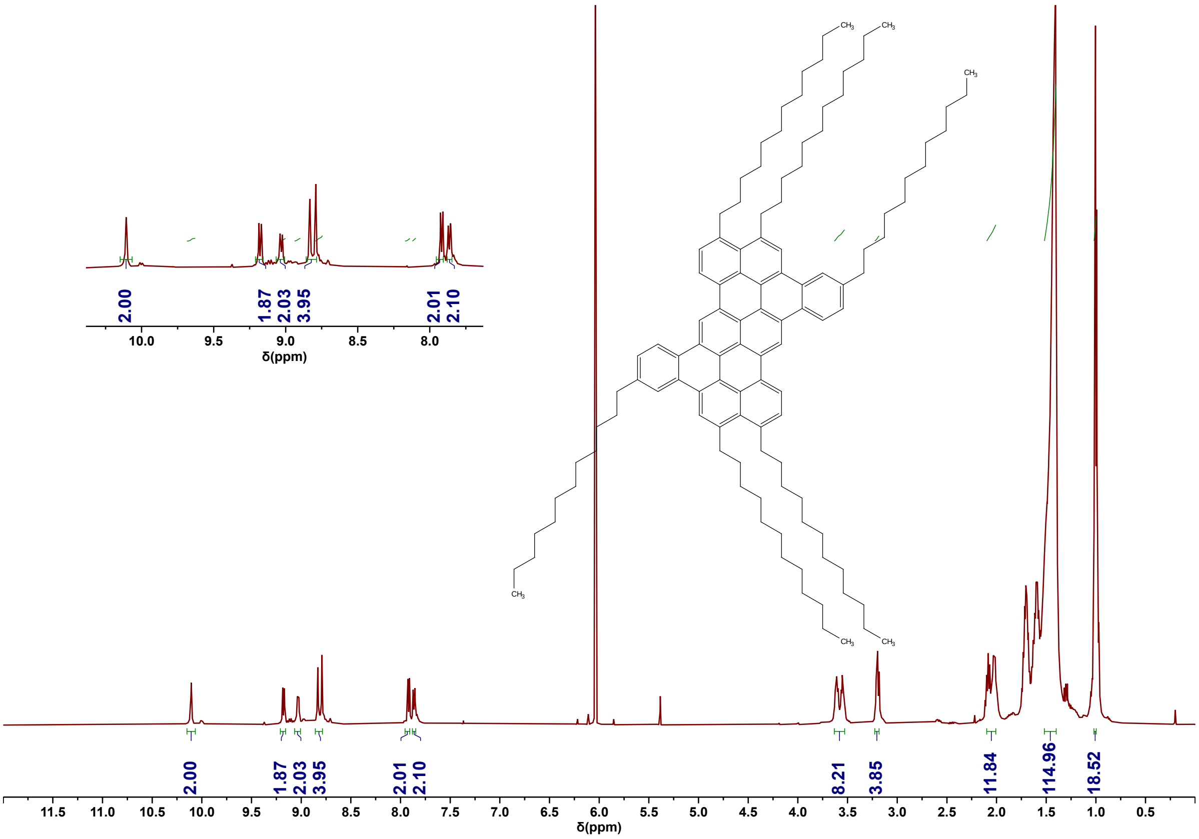

Figure S23. ^{1}H NMR spectrum of crude **PT12** recorded at 120 °C.

8. MALDI-TOF analysis of synthesized compounds

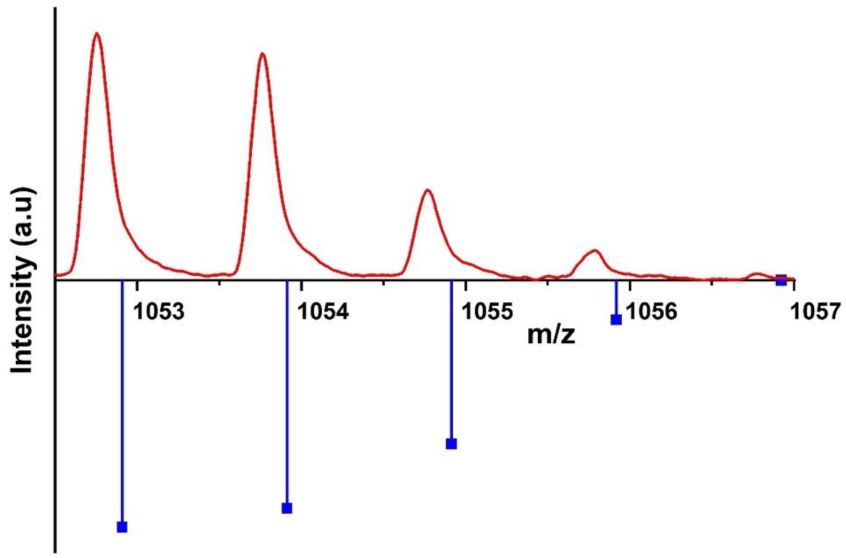

Figure S24. MALDI-TOF mass spectrum of **2** (red curve = experimental result, blue line = calculated result).

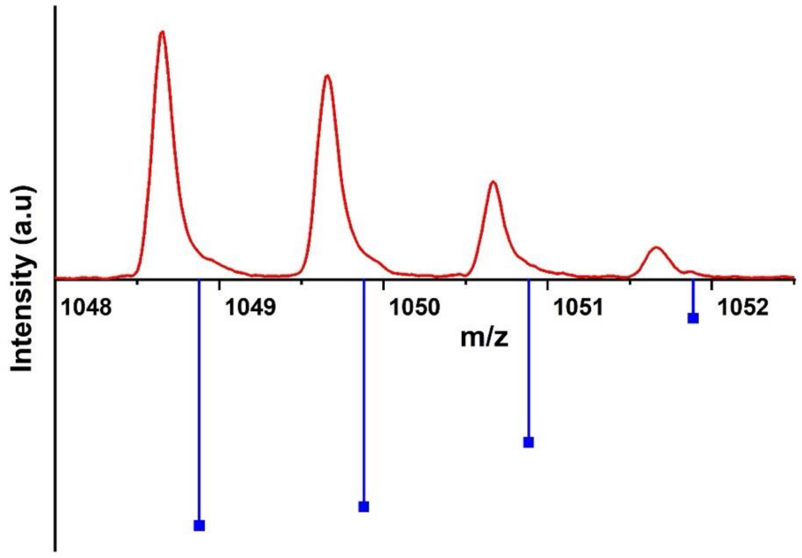

Figure S25. MALDI-TOF mass spectrum of **T12** (red curve = experimental result, blue line = calculated result).

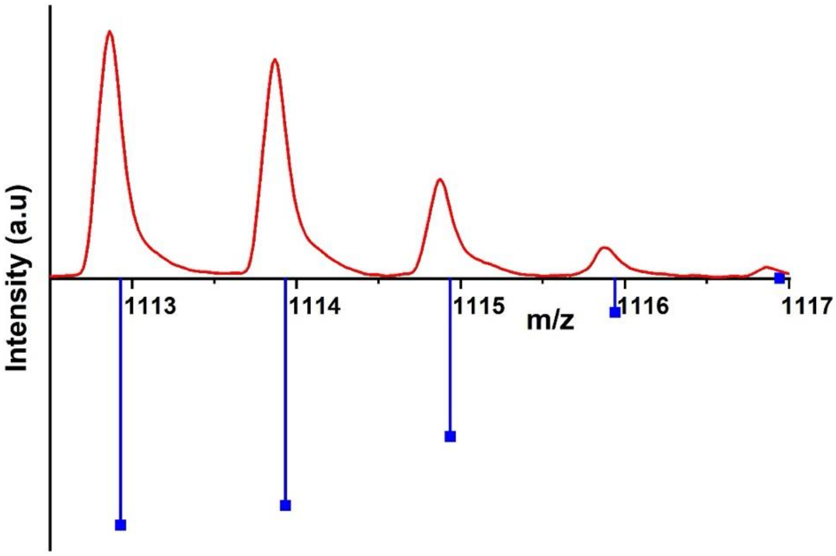

Figure S26. MALDI-TOF mass spectrum of **3** (red curve = experimental result, blue line = calculated result).

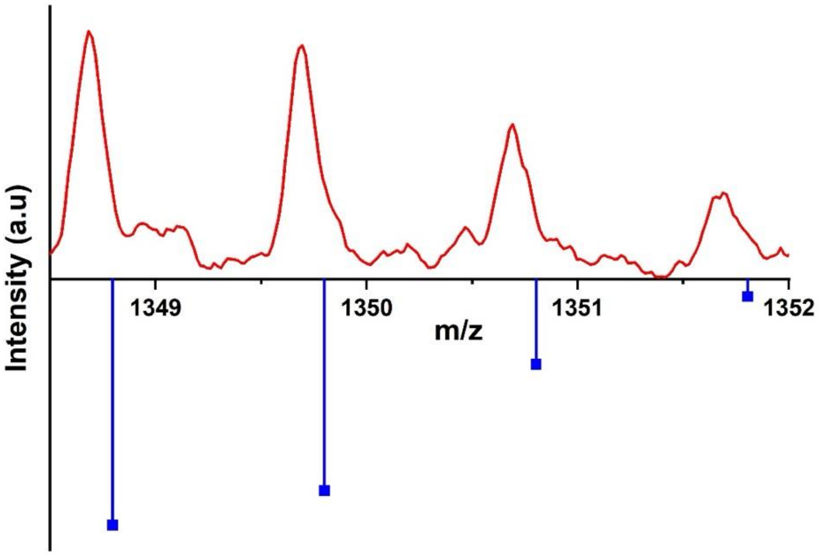

Figure S27. MALDI-TOF mass spectrum of **4** (red curve = experimental result, blue line = calculated result).

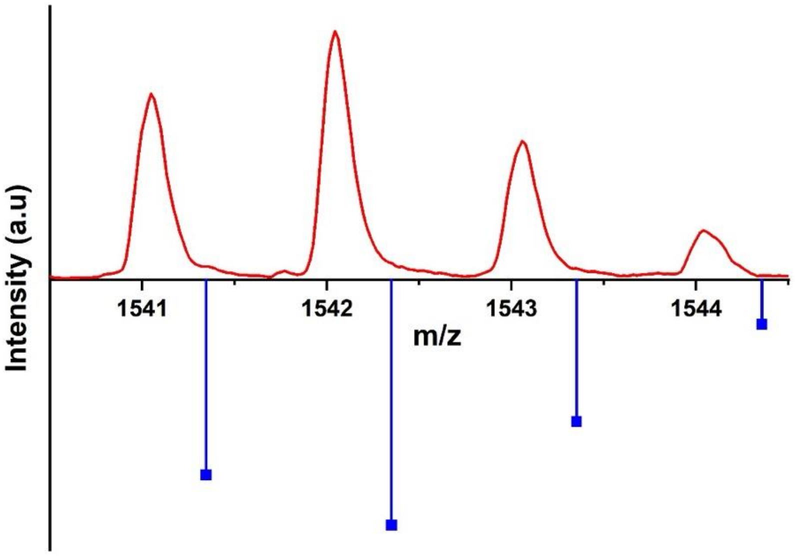

Figure S28. MALDI-TOF mass spectrum of **5** (red curve = experimental result, blue line = calculated result).

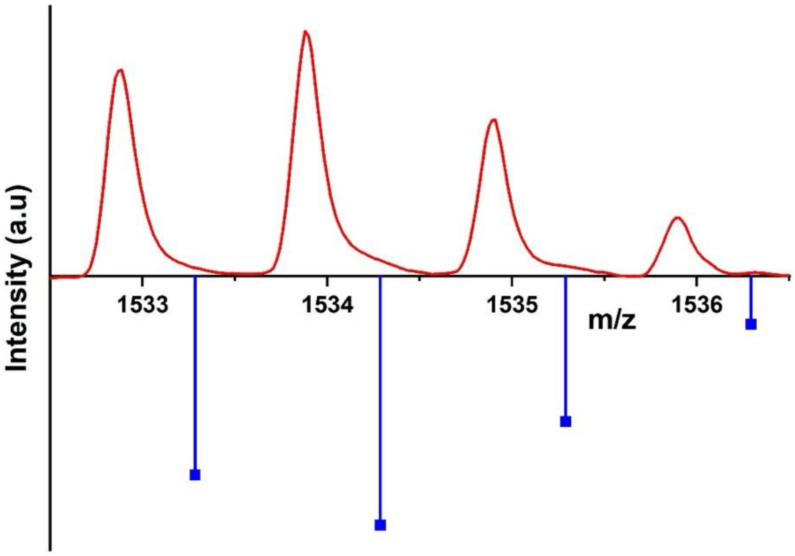

Figure S29. MALDI-TOF mass spectrum of **PT12** (red curve = experimental result, blue line = calculated result).

8. References:

[44] G. W. T. M. J. Frisch, H. B. Schlegel, G. E. Scuseria, M. A. Robb, J. R. Cheeseman, G. Scalmani, V. Barone, B. Mennucci, G. A. Petersson, H. Nakatsuji, M. Caricato, X. Li, H. P. Hratchian, A. F. Izmaylov, J. Bloino, G. Zheng, J. L. Sonnenberg, M. Hada, M. Ehara, K. Toyota, R. Fukuda, J. Hasegawa, M. Ishida, T. Nakajima, Y. Honda, O. Kitao, H. Nakai, T. Vreven, J. A. Montgomery, Jr., J. E. Peralta, F. Ogliaro, M. Bearpark, J. J. Heyd, E. Brothers, K. N. Kudin, V. N. Staroverov, R. Kobayashi, J. Normand, K. Raghavachari, A. Rendell, J. C. Burant, S. S. Iyengar, J. Tomasi, M. Cossi, N. Rega, J. M. Millam, M. Klene, J. E. Knox, J. B. Cross, V. Bakken, C. Adamo, J. Jaramillo, R. Gomperts, R. E. Stratmann, O. Yazyev, A. J. Austin, R. Cammi, C. Pomelli, J. W. Ochterski, R. L. Martin, K. Morokuma, V. G. Zakrzewski, G. A. Voth, P. Salvador, J. J. Dannenberg, S. Dapprich, A. D. Daniels, O. Farkas, J. B. Foresman, J. V. Ortiz, J. Cioslowski, and D. J. Fox, *Vol. Revision D.01* (Ed.: W. CT), Gaussian, Inc., **2013**.

[45] R. Dennington, T. Keith, J. Millam, *Semichem Inc.*, *Shawnee Mission KS, GaussView, Version 5, 2009.*
